# Rat hepatitis E virus (*Rocahepevirus ratti*) in people living with HIV

**DOI:** 10.1080/22221751.2023.2295389

**Published:** 2023-12-14

**Authors:** María Casares-Jimenez, Antonio Rivero-Juarez, Pedro Lopez-Lopez, María Luisa Montes, Roser Navarro-Soler, Joaquín Peraire, Nuria Espinosa, María Remedios Alemán-Valls, Tránsito Garcia-Garcia, Javier Caballero-Gomez, Diana Corona-Mata, Ignacio Perez-Valero, Rainer G. Ulrich, Antonio Rivero

**Affiliations:** a Infectious Diseases Unit, Reina Sofia University Hospital, Maimonides Instituto for Biomedical Research (IMIBIC), University of Cordoba (UCO), Cordoba, Spain; b CIBERINFEC, ISCIII – CIBER on Infectious Diseases, Carlos III Health Institute, Madrid, Spain; c HIV Unit, Internal Medicine Service, La Paz University Hospital, IdiPAZ, Madrid, Spain; d Infectious Diseases Unit, 12 de Octubre University Hospital, Madrid, Spain; e Infectious Diseases Unit, Joan XXIII University Hospital, IISPV, Rovira i Virgili University, Tarragona, Spain; f Infectious Diseases and Clinical Microbiology Unit, Virgen del Rocío University Hospital, CSIC, IbIS, University of Seville, Seville, Spain; g Infectious Diseases Unit, Canarias University Hospital, La Laguna, Spain; h Immunogenomic and Molecular Pathogenesis, Zoonoses and Emerging diseases Unit (ENZOEM), Genetic Department, University of Cordoba, Cordoba, Spain; i Animal Health Unit, Zoonoses and Emerging diseases Unit (ENZOEM), University of Cordoba, Cordoba, Spain; j Institute of Novel and Emerging Infectious Diseases, Friedrich-Loeffler-Institut, Federal Research Institute for Animal Health, Greifswald-Insel Riems, Germany; k German Centre for Infection Research (DZIF), partner site Hamburg-Lübeck-Borstel-Riems, Greifswald-Insel Riems, Germany

**Keywords:** Hepatitis E, rat hepatitis E virus, acute hepatitis, HIV, Zoonoses, public health

## Abstract

Rat hepatitis E virus (ratHEV; species *Rocahepevirus ratti*) is considered a newly emerging cause of acute hepatitis of zoonotic origin. ratHEV infection of people living with HIV (PLWH) might portend a worse, as with hepatitis E virus (HEV; species *Paslahepevirus balayani*), and consequently this group may constitute a high-risk population. We aimed to evaluate the prevalence of ratHEV by measuring viral RNA and specific IgG antibodies in a large Spanish cohort of PLWH. Multicentre study conducted in Spain evaluating PLWHIV included in the Spanish AIDS Research Network (CoRIS). Patients were evaluated for ratHEV infection using PCR at baseline and anti-ratHEV IgG by dot blot analysis to evaluate exposure to ratHEV strains. Patients with detectable ratHEV RNA were followed-up to evaluate persistence of viremia and IgG seroconversion. Eight-hundred and forty-two individuals were tested. A total of 9 individuals showed specific IgG antibodies against ratHEV, supposing a prevalence of 1.1 (95% CI; 0.5%−2.1%). Of these, only one was reactive to HEV IgG antibodies by ELISA. One sample was positive for ratHEV RNA (prevalence of infection: 0.1%; 95% CI: 0.08%−0.7%). The case was a man who had sex with men exhibiting a slightly increased alanine transaminase level (49 IU/L) as only biochemical alteration. In the follow-up, the patients showed undetectable ratHEV RNA and seroconversion to specific ratHEV IgG antibodies. Our study shows that ratHEV is geographical broadly distributed in Spain, representing a potential zoonotic threat.

## Introduction

The *Hepeviridae* family is composed of four genera with different hosts, and two of the genera are considered zoonotic threats [1]. Among those species affecting humans, *Paslahepevirus balanyani*, also known as hepatitis E virus (HEV), represents a major health threat [2] and is ranked as the leading cause of acute viral hepatitis worldwide [3]. HEV genotypes 3, 4 and 7 are zoonotic, exhibiting a global distribution, and are linked to the consumption of raw or undercooked meat or milk from multiple mammal species [4]. Recently, the zoonotic potential of rat hepatitis E virus (ratHEV, species *Rocahepevirus ratti*) [5], whose main host is rodents, has been recognized [6]. Because ratHEV is considered a newly emerging viral zoonotic disease, very little is known about the virus in the context of human infection.

The first human cases of ratHEV infection were reported in 2018 in Hon-Kong [7], followed by reports in Canada [8], Spain [9], and, very recently, France [10]. Despite the low number of cases reported, its clinical course seems to be similar to HEV [11], ranging from moderate to severe acute hepatitis that can be fatal in patients with severe underlying disease, such as metastatic cancer [9]. Similarly, the development of chronic ratHEV infection has also been documented in immunosuppressed patients [7, 10]. Because of the scarcity of studies evaluating the prevalence of this emerging virus, which are mainly focus on only two subsets of patients (such as transplant recipients and patients with acute hepatitis) [7, 9–13], the epidemiology and clinical impact of the infection in other highly vulnerable populations is completely unknown. For example, ratHEV infection in people living with HIV (PLWH) might portend a worse prognosis as with HEV [14]. The worse prognosis is due to two reasons: the underlying immunosuppression that could trigger the development of chronic infection [5], and the high rate of severe underlying disorders that could worsen prognosis [15]. For this reason, studies specifically evaluating ratHEV infection in PLWH are needed.

Molecular assays are not the proper way to study the circulation of the virus as viremia is usually cleared in the short term. The absence of commercial serological assays for the screening of specific ratHEV antibodies significantly limits our understanding of its epidemiology because viral circulation or associated risk factors cannot be evaluated in large-scale populations. In addition, serological investigations by ELISA or blot formats may be limited in their specificity due to high-cross-reactivity of viral antigens and existence of still unknown viruses.

For these reasons, our study aimed to evaluate the prevalence of ratHEV by measuring viral RNA and specific IgG antibodies in a large Spanish cohort of PLWH.

## Methods

### Study population

**Figure 1.**
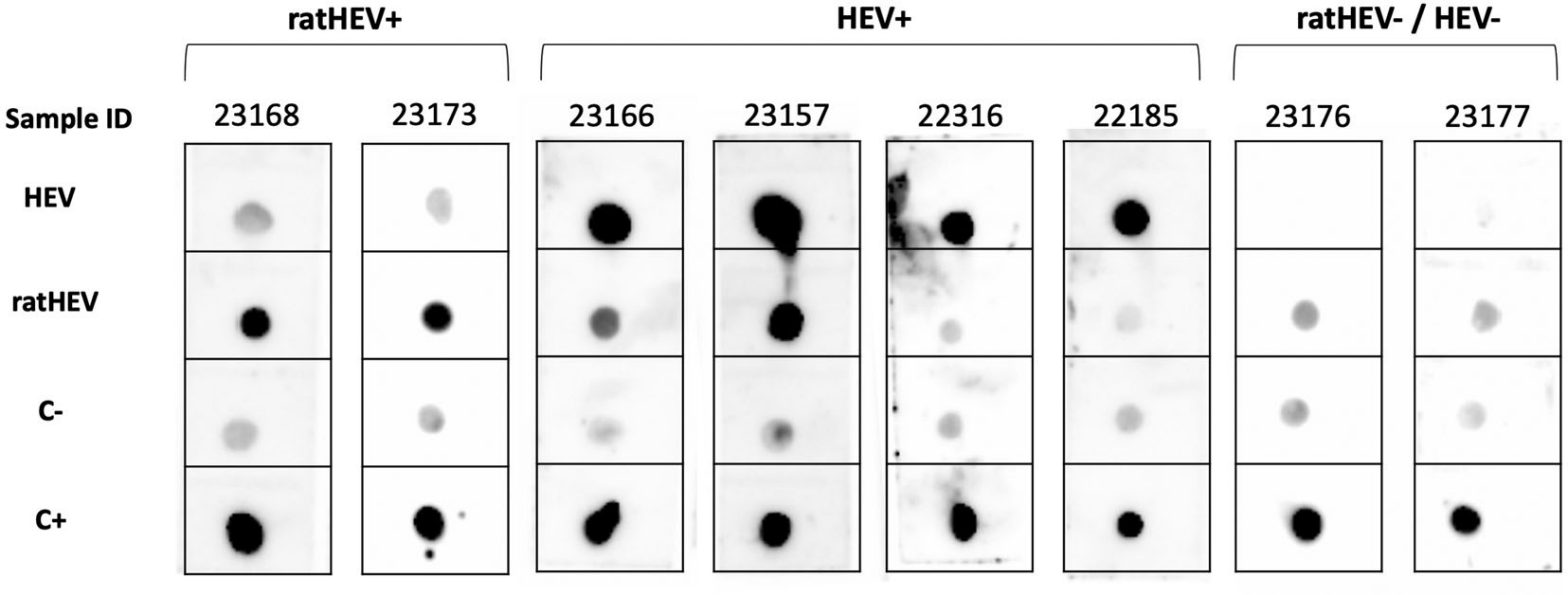
Validation of the dot blot for the detection of HEV genotype 3 and ratHEV IgG specific antibodies. Carboxy-terminal segments of the capsid proteins were used as antigen for HEV genotype 3 and ratHEV in the first and second spot (HEV and ratHEV), respectively. As negative control (C-) a nucleocapsid protein derivative of *Puumala orthohantavirus* strain Vranica/Hällnäs was used. As positive control, we used an E. coli M15 lysate (C+).

The cohort of adult PLWH of the AIDS Research Network (CoRIS) is an open, prospective, multicentre cohort of adult subjects with confirmed HIV infection launched in 2004, including those who are naïve to antiretroviral therapy (ART) at cohort entry and who are recruited to HIV-1 and HIV-2 care units of the Spanish Public Health System [16], which represents the standard place of treatment for the great majority of persons in Spain. All centres recruited all subjects seen for the first time who met the following criteria: confirmed HIV diagnosis and naïve to ART. The cohort is linked to a centralized BioBank, where patients’ blood samples were processed and cryopreserved immediately after reception and then stored at −80°C [17]. The BioBank obtained the UNE-EN-ISO 9001:2008 Systems of Quality Management Requirements. For the proposed study, we analyzed samples from patients recruited in a previous study where prevalence and incidence of HEV was analyzed [18]. This population was composed by 842 individuals, randomly selected by the CoRIS and Biobank coordinators from 8.468 patients, at follow-up in 28 hospitals belonging to 13 Spanish cities (Madrid, Tarragona, Valencia, Granada, Barcelona, Mallorca, Seville, Alicante, Gijon, Elche, Gran Canarias, Malaga, Murcia, San Sebastian, Badalona, and Corunna) and with available serum samples in the centralized Biobank in 2014 (Figure 1).

### Serological evaluation of ratHEV

All patients were previously tested for IgG HEV antibodies by ELISA using the Wantai Diagnostic IgG kit (Beijing, China) [18]. In the current study, all patients were tested for ratHEV IgG antibodies by dot blot (DB) to evaluate exposure to either HEV or ratHEV strains, including HEV genotype 3 and ratHEV. Carboxy-terminal segments of the capsid proteins were used as antigen for HEV genotype 3 and ratHEV. As negative control a nucleocapsid protein derivative of *Puumala orthohantavirus* strain Vranica/Hällnäs was used. As positive control, we used an E. coli M15 lysate. This bacterial culture of competent cells include the pREP4 plasmid that confers kanamycin resistance and constitutively expresses the lac repressor protein encoded by the lac I gene. Recombinant proteins were produced as His-tagged in *Escherichia coli* and purified by nickel-chelate affinity chromatography [19, 20]. Then, purified proteins were run on a 12% SDS‒PAGE gel, transferred to a polyvinylidene difluoride (PVDF) membrane and analyzed for control by anti-His tag and HEV capsid protein cross-reactive monoclonal antibodies (Merck, Darmstadt, Germany) [21]. For DB, 5 µl (1 µg) of each purified protein was spotted onto an activated PVDF membrane. The membrane was washed in H_2_O, blocked for 1 h in TBS-T (20 mM Tris-HCl, 150 mM NaCl, 0,1% Tween 20, pH 7,5) containing 5% skimmed milk. Serum samples were diluted 1:100 in 5% skimmed milk in TBS-T, incubated overnight, and the antigen–antibody reaction was detected by adding purified recombinant protein A/G conjugated with horseradish peroxidase (HRP) (Thermo Scientific, Schwerte, Germany) diluted 1:1000 in 5% TBS-T. The immunoreaction was detected using Clarity Western ECL Substrate (Bio-Rad, Feldkirchen, Germany) and documented in a ChemiDoc^TM^ MP Imaging System (Bio-Rad) with an exposure time between one and 60 s. Seropositivity was confirmed when compatible dot was observed against ratHEV antigens and not dots against HEV.

For the validation of the assay, we used three sets of samples: (i) Patients with confirmed ratHEV infection by PCR (n = 2) [9], (ii) Patients with confirmed HEV infection by PCR and showing IgG antibodies by ELISA (n = 4) [22], and (iii) Patients diagnosed with acute hepatitis with negative HEV and ratHEV RNA and HEV IgM and IgG antibodies for HEV by ELISA (n = 2) (Figure 1). Both individuals with ratHEV infection exhibit a specific dot on the ratHEV antigen. Three out of four patients with HEV infection only show specific dots on HEV genotype 3 antigens, while one shows dots on both HEV and ratHEV antigens, suggesting cross-reaction. Both negative control individuals only show dots on the positive control position. The DB analysis was performed centrally in the Clinical Virology and Zoonoses lab of the IMIBIC after agreement with the Institute of Novel and Emerging Infectious Diseases, Friedrich-Loeffler-Institut, (Germany).

### Molecular evaluation for ratHEV infection

All patients were evaluated for ratHEV infection using broad-spectrum nested RT–PCR targeting the RdRp gene, as described previously [18]. RNA was extracted from 400 µl of serum using the QIAamp Mini Elute virus spin kit (Qiagen, Hilden, Germany) and QIAcube (Qiagen, Hilden, Germany), eluting the purified RNA in 50 µl of AVE buffer (Qiagen, Hilden, Germany). Ten microliters of RNA were used in combination with the One-Step RT–PCR kit (Qiagen, Hilden, Germany) for the reverse transcription and first round of PCR. For the second round, 5 µL of the first PCR product in combination with the Promega Master Mix (Madison, WI USA) was used. The 1st World Health Organization (WHO) International Standard for HEV RNA Nucleic Acid Amplification Techniques (NAT)-Based Assays, consistent with HEV subgenotype 3a, provided by the Paul-Ehrlich-Institut (PEI code 6219/10) was used as a positive control.

The amplicons were examined on 1.5% agarose gels stained with RedSafe™ Nucleic Acid Staining solution. PCR products with the correct target size (approximately 330 nucleotides) were purified using Illustra™ ExoProStar™. Both sense strands were sequenced using a BigDye Terminator cycle sequencing ready reaction kit on an ABI Prism 3100 genetic analyzer (Applied Biosystems, Foster City, CA, USA). For taxonomic assignment within the *Hepeviridae* family, we used the HEVnet genotyping tool (https://www.rivm.nl/mpf/typingtool/hev/) and confirmed the results by Basic Local Alignment Search Tool (BLAST) analysis. The molecular analysis was performed centrally in the Clinical Virology and Zoonoses lab of the Instituto Maimonides de Investigación Biomédica de Córdoba (IMIBIC), Spain.

## Statistical analysis

The main outcome variable was the presence of ratHEV IgG antibodies. The prevalence of ratHEV infection was calculated, providing a two-sided 95% confidence interval (95% CI) using the exact binomial distribution. The proportion of individual with specific ratHEV IgG reactive to HEV IgG antibodies by ELISA were reported. The secondary outcome variable was the presence of ratHEV RNA in serum.

### Ethics statement

This study was designed and conducted in accordance with the Declaration of Helsinki. The local and national Clinical Trial and Ethical Committee approved the study protocol (protocol reference number 5081). Each CoRIS participant provided his or her written informed consent prior to enrolling in this study. The CoRIS cohort was approved by the Research Ethics Committee of the Gregorio Marañón Hospital.

### Data availability

All data generated or analyzed during the study are included in the article. The datasets used and/or analyzed during the present research project are available from the corresponding author upon reasonable request. The obtained ratHEV sequence was submitted to GenBank (accession number: OP793791).

## Results

### Study population

**Figure 2.**
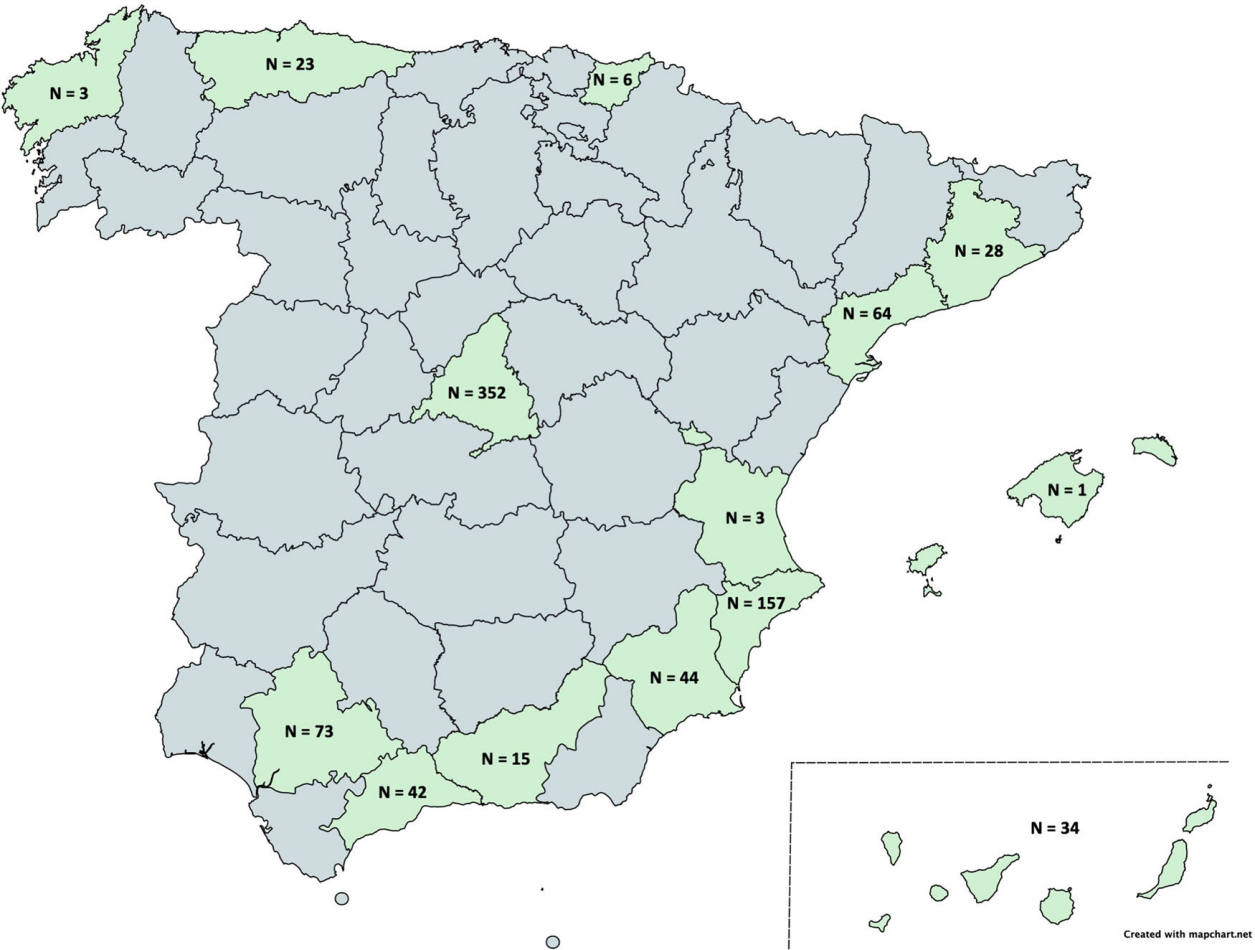
Geographical distribution of patients tested for ratHEV RNA and IgG specific antibodies.

A total of 842 individuals were included in the study. The detailed study participant demographics can be found elsewhere [14]. Briefly, the majority of patients were male (n = 747; 88.8%), with a median age of 36.3 years (SD = 15.6 years). The geographical distribution of the samples tested is presented in Figure 2.

**Figure 3.**
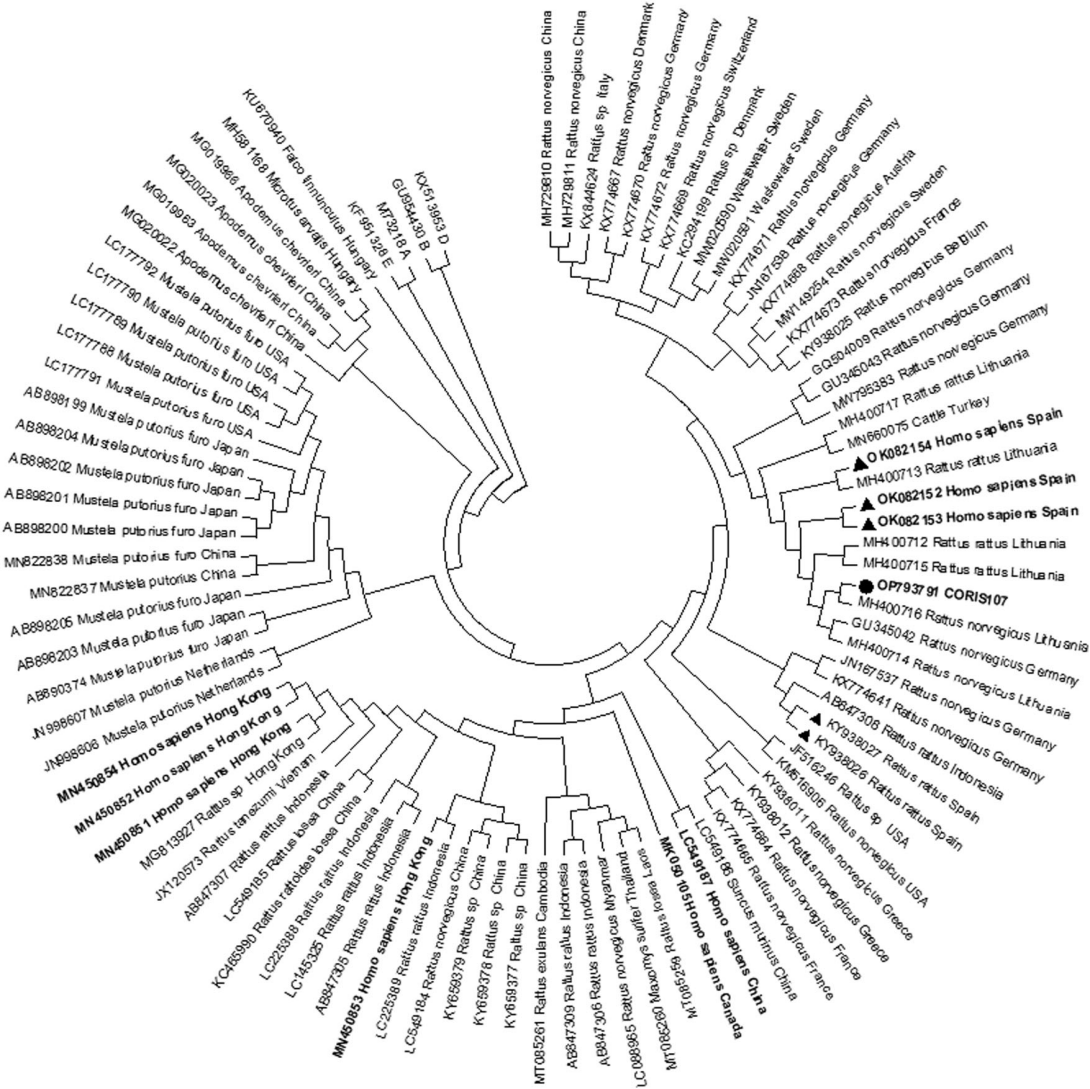
Phylogenetic analysis of the sequence identified in the study. Sequence of the patient identified in the present study is marked with a circle (•), human cases and rodents’ strains identified previously in Spain with a triangle (▴). In bold its highlighted strains identified in humans. The evolutionary history was inferred by using the Maximum Likelihood method based on the Tamura-Nei model. The bootstrap consensus tree inferred from 1,500 replicates is taken to represent the evolutionary history of the taxa analyzed. Branches corresponding to partitions reproduced in less than 50% bootstrap replicates are collapsed. Initial tree(s) for the heuristic search were obtained automatically by applying Neighbor-Join and BioNJ algorithms to a matrix of pairwise distances estimated using the Maximum Composite Likelihood (MCL) approach, and then selecting the topology with superior log likelihood value. The analysis involved 102 nucleotide sequences. Codon positions included were 1st + 2nd + 3rd + Noncoding. All positions containing gaps and missing data were eliminated. There are a total of 221 positions in the final dataset.

### Serological evaluation of ratHEV

**Figure 4.**
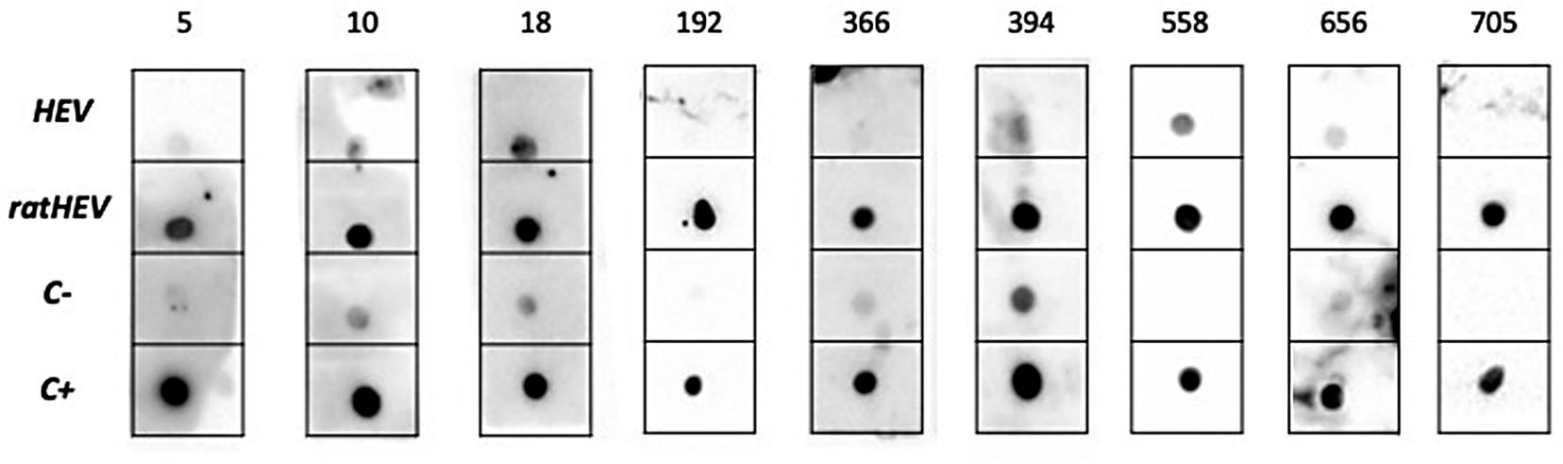
DB results for the 9 positive ratHEV IgG antibodies sera. The numbers correspond to the ID of the sample.

A total of 9 patients exhibited specific IgG antibodies against ratHEV by DB (Figure 4), supposing a prevalence of 1.1 (95% CI; 0.5%−2.1%). Demographic characteristics of patients with anti-ratHEV IgG antibodies is shown in Table 1. It was detected at least one patient with positive antibodies in 6 out 13 cities evaluated (46.1%), suggesting a wide national distribution of the virus. DB results for all patients are shown as technical annexe.

**Table 1.** Characteristics of patients exhibiting specific ratHEV IgG antibodies.

Patient ID	Gender	Age	Origin	Residence	HEV IgG by ELISA
5*	Male	52	Spain	Madrid	Yes
10	Male	52	Spain	Elche	No
18	Male	47	Spain	Madrid	No
192	Male	42	Spain	Elche	No
366	Male	44	Spain	Elche	No
394	Female	57	Spain	Elche	No
558	Male	33	Spain	Alicante	No
686	Male	41	Spain	Madrid	No
705	Male	45	Spain	Canary Island	No

*This patient seroconverted after diagnosis of acute infection.

Among the patients with specific ratHEV IgG antibodies, just one (11.1%) showed HEV IgG antibodies by ELISA (Table 1).

### Molecular evaluation of ratHEV

One sample was positive for ratHEV RNA, suggesting a prevalence of infection of 0.1%; (95% CI: 0.08%−0.7%). The sequence determined (GenBank accession number OP793791) was consistent with ratHEV and phylogenetically related to strains previously identified patients from Spain, and strains detected in rats from different European countries (Figure 3). The case was a man who had sex with men on ART with an undetectable HIV viral load and 368 CD4 + cells/mm^3^, with serological markers of past hepatitis B virus (HBV) infection (positive for HBV anti-core and anti-surface antigen (HBsAg) antibodies). The patient exhibited a slightly increased alanine transaminase (ALT) level (49 IU/L) as only biochemical alteration. The patient lives in Madrid (central Spain) and did not report contact with animals or travel in the previous months. Neither IgG nor IgM antibodies against HEV were detected in this sample by ELISA. In the most recent sample after diagnosis (16 months later), the ratHEV RNA could not be detected, suggesting a self-limited course of the infection. At this point, HEV-reactive IgG antibodies were detected by ELISA, indicating a seroconversion. After DB analysis, we confirmed that IgG antibodies were specific for ratHEV. The CD4 + cell count at this point was 476 cells/mm^3^.

## Discussion

Because of its recent recognition as a zoonotic agent, epidemiological data about ratHEV are scarce and are limited to clinical observations, without large population surveillance. This study is the first large-scale cohort study specifically designed to evaluate the presence and circulation of ratHEV in a high-risk population, PLWHIV.

The determination of IgG antibodies is an outstanding tool to evaluate viral circulation. Nevertheless, there are no available commercial screening tests for the detection of specific ratHEV antibodies. Here, we use a made-in-house DB for the determination of both HEV- and ratHEV-specific IgG antibodies in individuals bearing anti-HEV IgG antibodies detected by ELISA. This may represent a favourable approach to evaluate the circulation of this emerging virus in asymptomatic individuals. In our study, 1.1% of patients were positive for ratHEV specific antibodies. Furthermore, these individuals were from a different geographical origin, from North to South Spain, including Canary Islands. Together with the previous cases reported in Spain (two cases in southern Spain and another one in the north) [9], and the detection of ratHEV RNA in Southern Spain rats [23], these findings imply that ratHEV may be widely distributed and, consequently, supposes a zoonotic threat at the national level. On the other hand, cross-reaction between HEV and ratHEV IgG antibodies has been documented, challenging the evaluation of ratHEV seroprevalence in the absence of commercial screening test. Because DB is not a cost-effective tool to screening large population, alternative strategies should be evaluated. In Hong Kong, the approach used to optimize the identification of patients bearing specific ratHEV IgG antibodies was to test only those individuals bearing IgG HEV antibodies by ELISA [24]. Nevertheless, this approach has been only tested in Asian context (an hyperendemic area), so might not be effective in Europe. In our study, only one of individuals with specific ratHEV IgG antibodies could be detected by HEV ELISA. Therefore, the strategy applied in Asia cannot be used in Europe because it would miss a high proportion of people exposed to rat HEV who are seronegative to ELISA.

The number of ratHEV cases described worldwide is limited, representing approximately 30 individuals [8–11]. The clinical spectrum varies from acute self-limited infection to fatal acute liver failure and persistent infection in patients with underlying immunosuppression. This spectrum has been described in both Asian and European ratHEV strains [8–11]. Because we designed a population-based screening without focusing on a specific syndrome (such as acute hepatitis), we were able to identify other clinical forms of the infection. The case of acute ratHEV infection identified in our study exhibited a subclinical course, producing only a slight increase in ALT levels. Thus, our study suggests that ratHEV, as HEV, is not limited to clinically evident infection. On the other hand, only one case of ratHEV has previously been reported in PLWHIV [5]. This case was a forty-three-year-old male with a total CD4 + cell count of 66 cells/mm^3^ [5] who experienced a chronic form of the infection for at least 5 months after diagnosis [5]. This chronic form was previously described in individuals with other underlying immunosuppressive conditions, including transplant recipients [7]. The case identified here experienced a self-limited infection. Consequently, our study suggests that, as for HEV, only patients with severe immunosuppression may be at risk of developing a chronic infection.

The case identified in the present study did not report contact with animals, including rats and other rodents. These findings are consistent with previous reports where the vast majority of cases do not recognize direct contact with rodents [5, 9, 11]. Nevertheless, because the viral strain identified exhibits high sequence similarity with those identified in rats, the source of infection seems to be most likely rats and their excreta, but the route remains undetermined. Because the virus is shed in feces [25], surface or food contamination may be the transmission route. Nevertheless, because the virus has been identified in other species and because of evidence of exposure in other hosts [26–28], an alternative transmission route cannot be dismissed.

Several limitations should be noted. First, this is a retrospective analysis, and consequently, epidemiological data about exposure and risk practice for ratHEV cannot be fully recorded. Second, DB and WB are not the proper tool for surveillance-based population studies. Alternative assays allowing fast and sensitive screening are needed to evaluate the real extent of ratHEV in the general population. In this sense, a recent manuscript has described an EIA method for the specific detection of ratHEV IgG antibodies that which will be really useful for that [29]. Moreover, ELISA and blot assay formats are limited in their conclusions on the causative viral agent, as cross-reactivity of HEV-reactive antibodies might be induced by so far unknown ratHEV-related hepeviruses as discussed previously [19]. Finally, the number of cases found was lower than expected, so factors associated with human ratHEV infection cannot be determined.

In conclusion, our study found that ratHEV is geographical widespread in Spain, representing a zoonotic threat. In PLWHIV, ratHEV seems to be similar to HEV with respect to its clinical course. Cohort-based surveillance is needed to evaluate the circulation of the virus in other settings and populations to establish the true extent of this emerging virus. These investigations will profit in the future from more specific serological assays, like neutralization assays.

## Supplementary Material

Technical_Annex_new

## Data Availability

All of the data generated or analyzed during the study are included in the article. The datasets used and/or analyzed during the present research project are available from the corresponding author upon reasonable request. The viral sequence is available in GenBank under accession number OP79379.
