# Supplementary material for: Rat hepatitis E virus (Rocahepevirus ratti) in people living with HIV
Source: Emerg Microbes Infect. 2023 Dec 14;13(1):2295389. doi: 10.1080/22221751.2023.2295389 (PMC10763910; doi:10.1080/22221751.2023.2295389)
Supplement: Technical_Annex_new [file TEMI_A_2295389_SM3171.pdf]

APPENDIX

LEYEND:

Positive antibodies for **RHEV**

Positive antibodies for **HEV**

Positive antibodies for **RHEV** and **HEV**

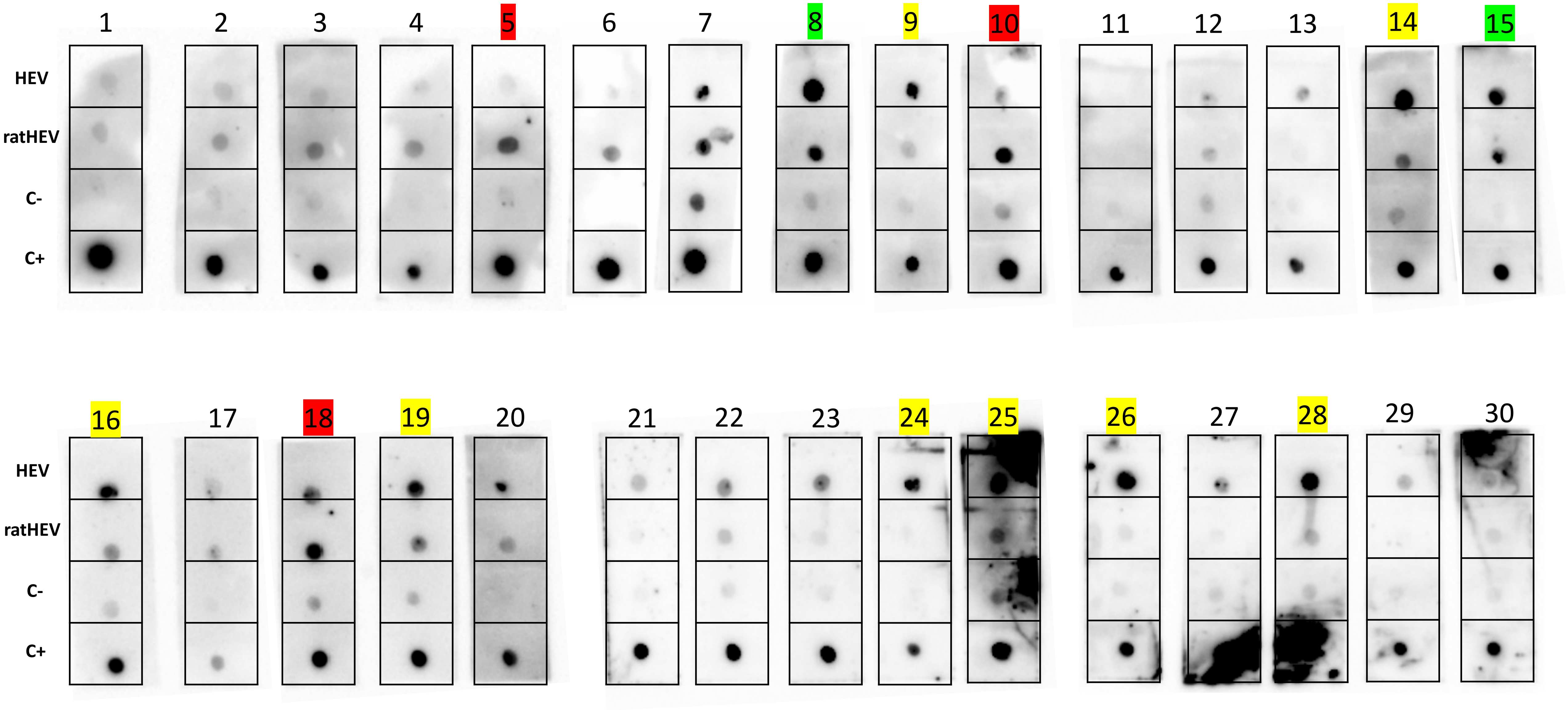
1
2
3
4
5
6
7
8
9
10
11
12
13
14
15
HEV
ratHEV
C-
C+
16
17
18
19
20
21
22
23
24
25
26
27
28
29
30
HEV
ratHEV
C-
C+

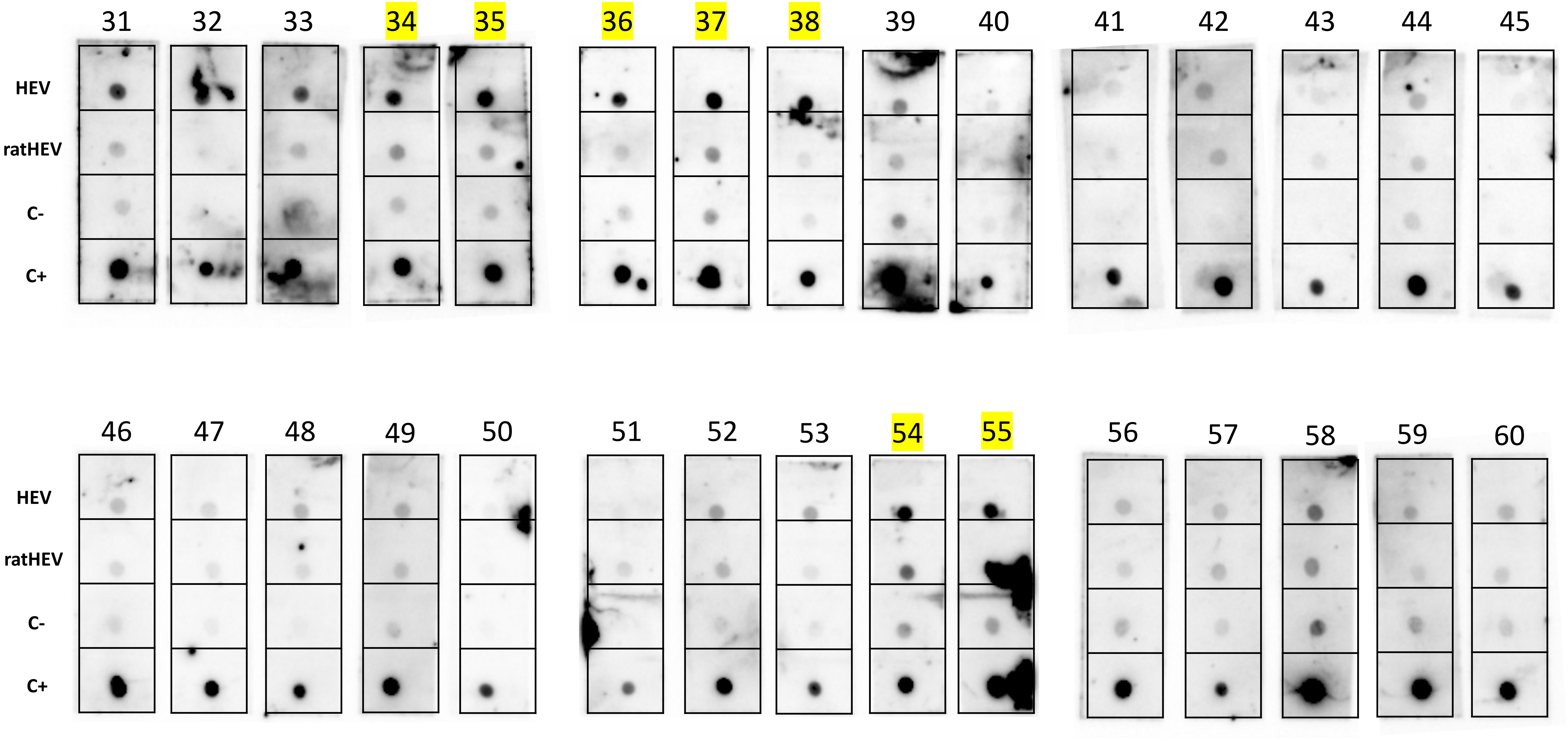
31 32 33 34 35 36 37 38 39 40 41 42 43 44 45
HEV
ratHEV
C-
C+
46 47 48 49 50 51 52 53 54 55 56 57 58 59 60
HEV
ratHEV
C-
C+

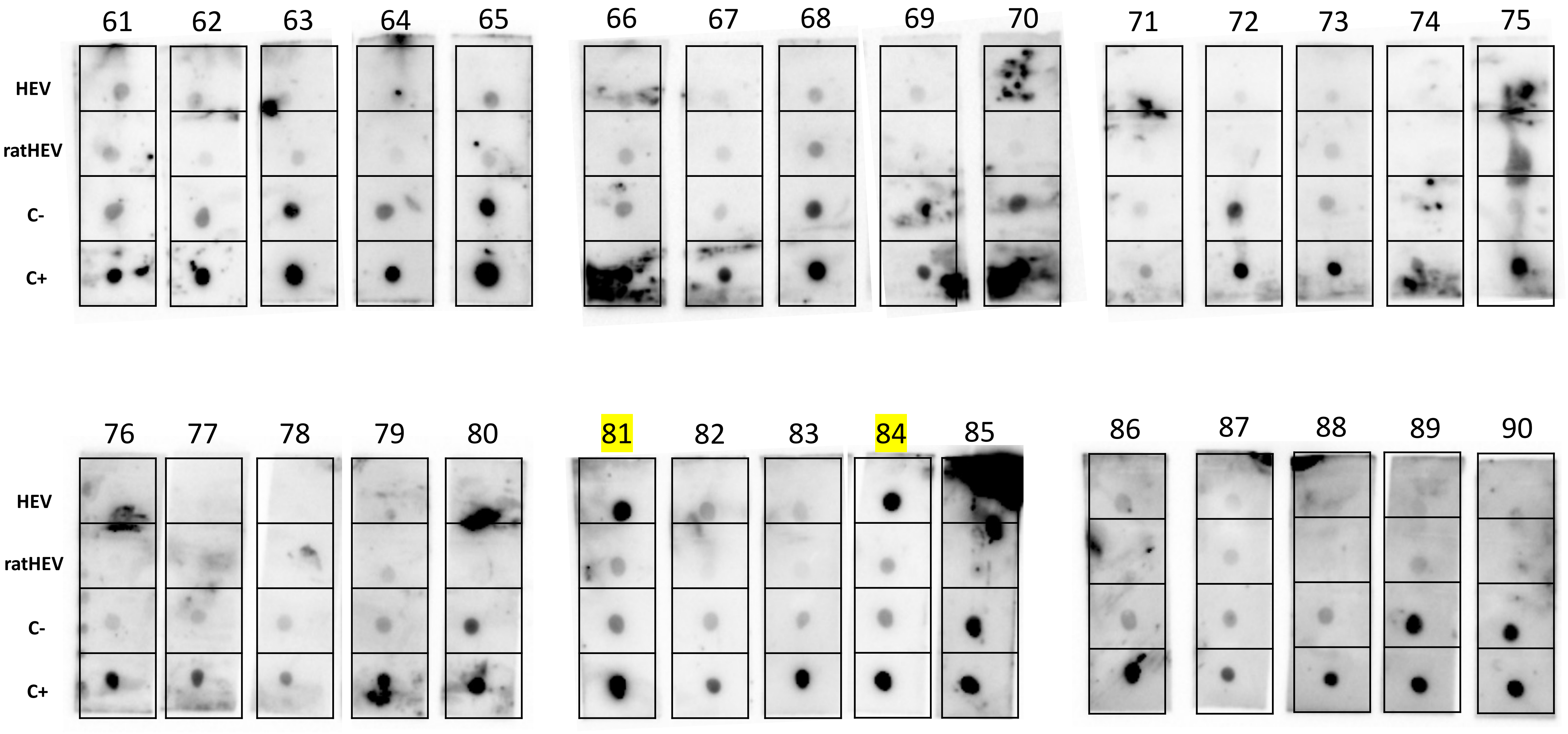
61
62
63
64
65
66
67
68
69
70
71
72
73
74
75
HEV
ratHEV
C-
C+
76
77
78
79
80
81
82
83
84
85
86
87
88
89
90
HEV
ratHEV
C-
C+

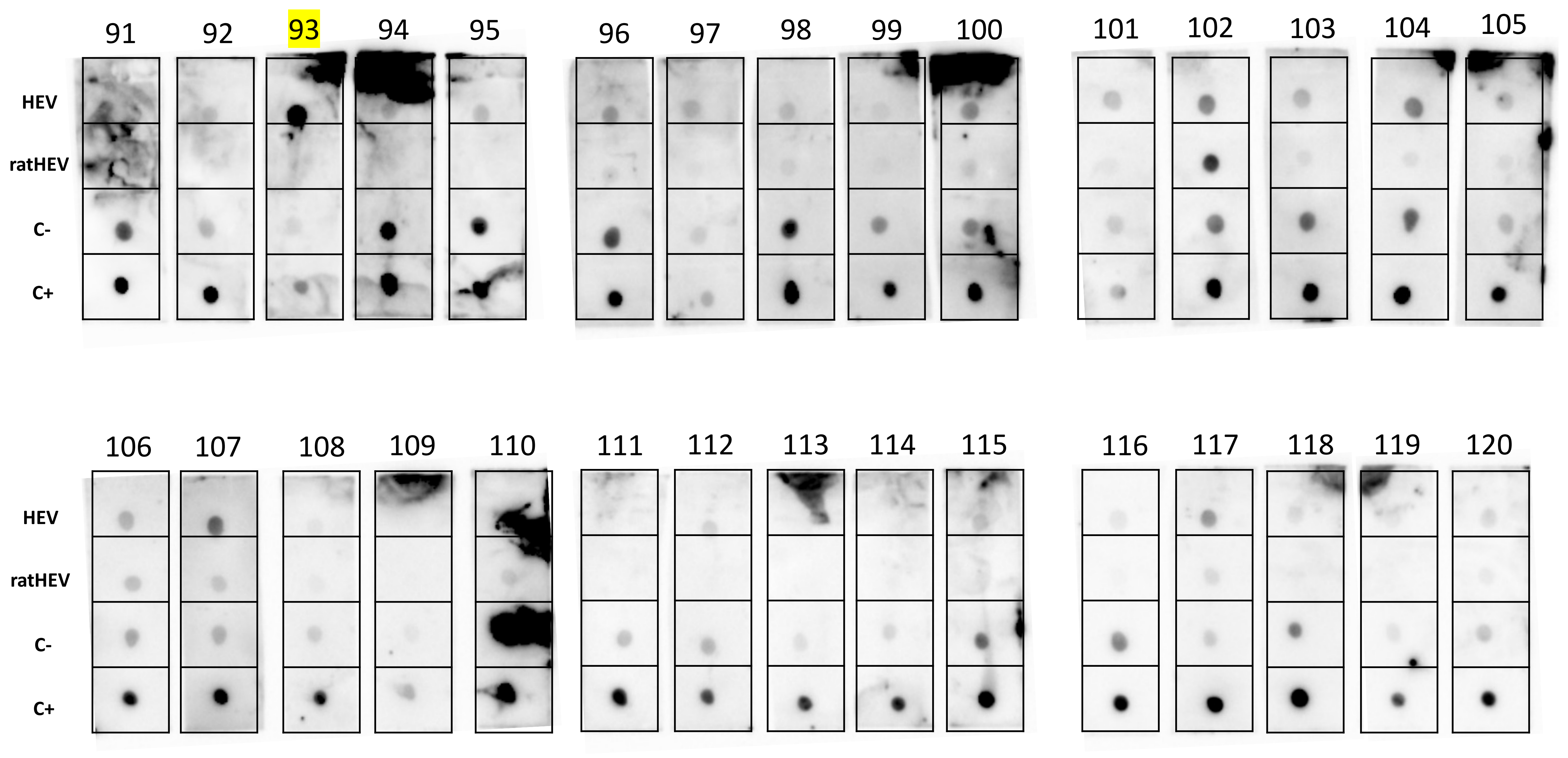

91
92
93
94
95
96
97
98
99
100
101
102
103
104
105
HEV
ratHEV
C-
C+
106
107
108
109
110
111
112
113
114
115
116
117
118
119
120
HEV
ratHEV
C-
C+

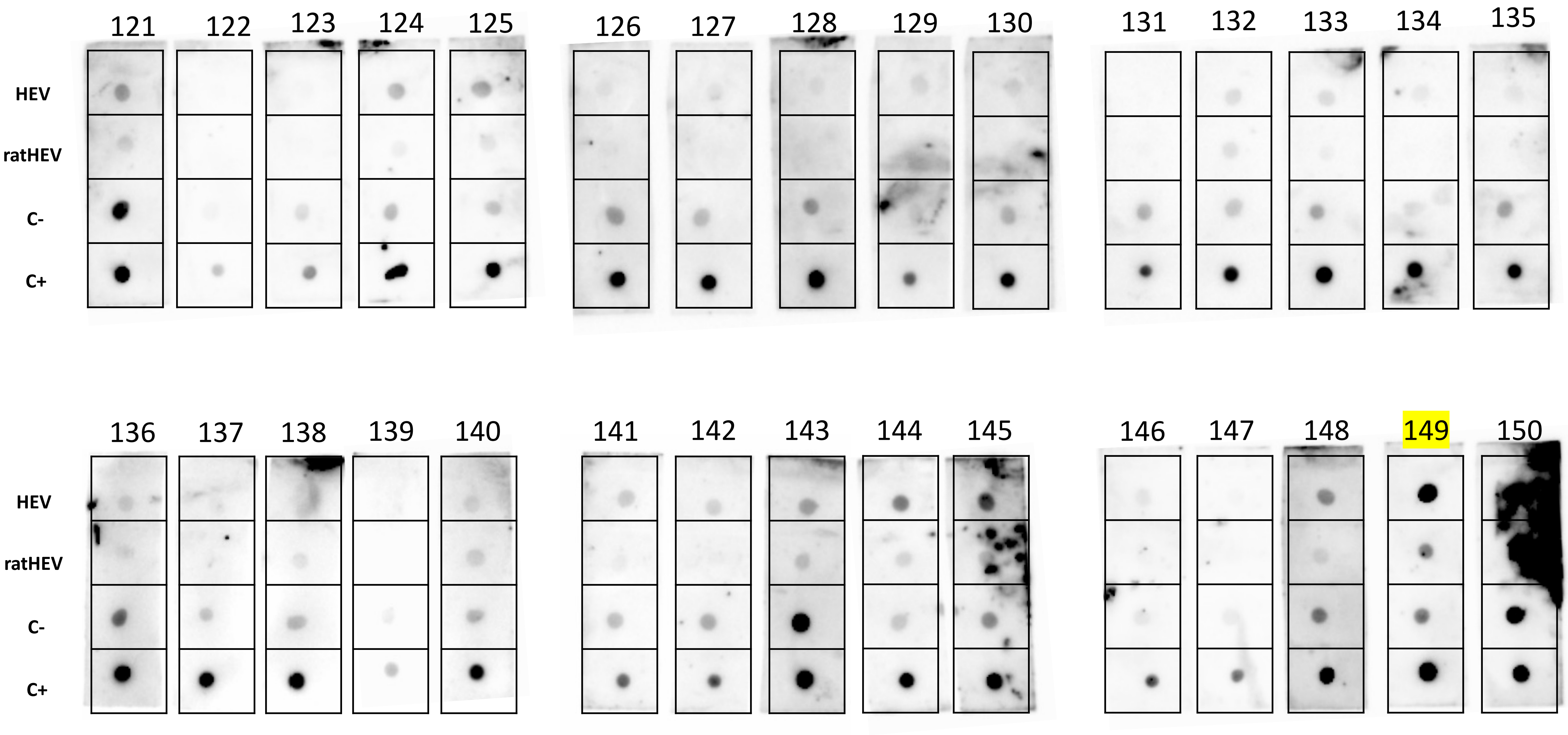
121
122
123
124
125
126
127
128
129
130
131
132
133
134
135
HEV
ratHEV
C-
C+
136
137
138
139
140
141
142
143
144
145
146
147
148
149
150
HEV
ratHEV
C-
C+

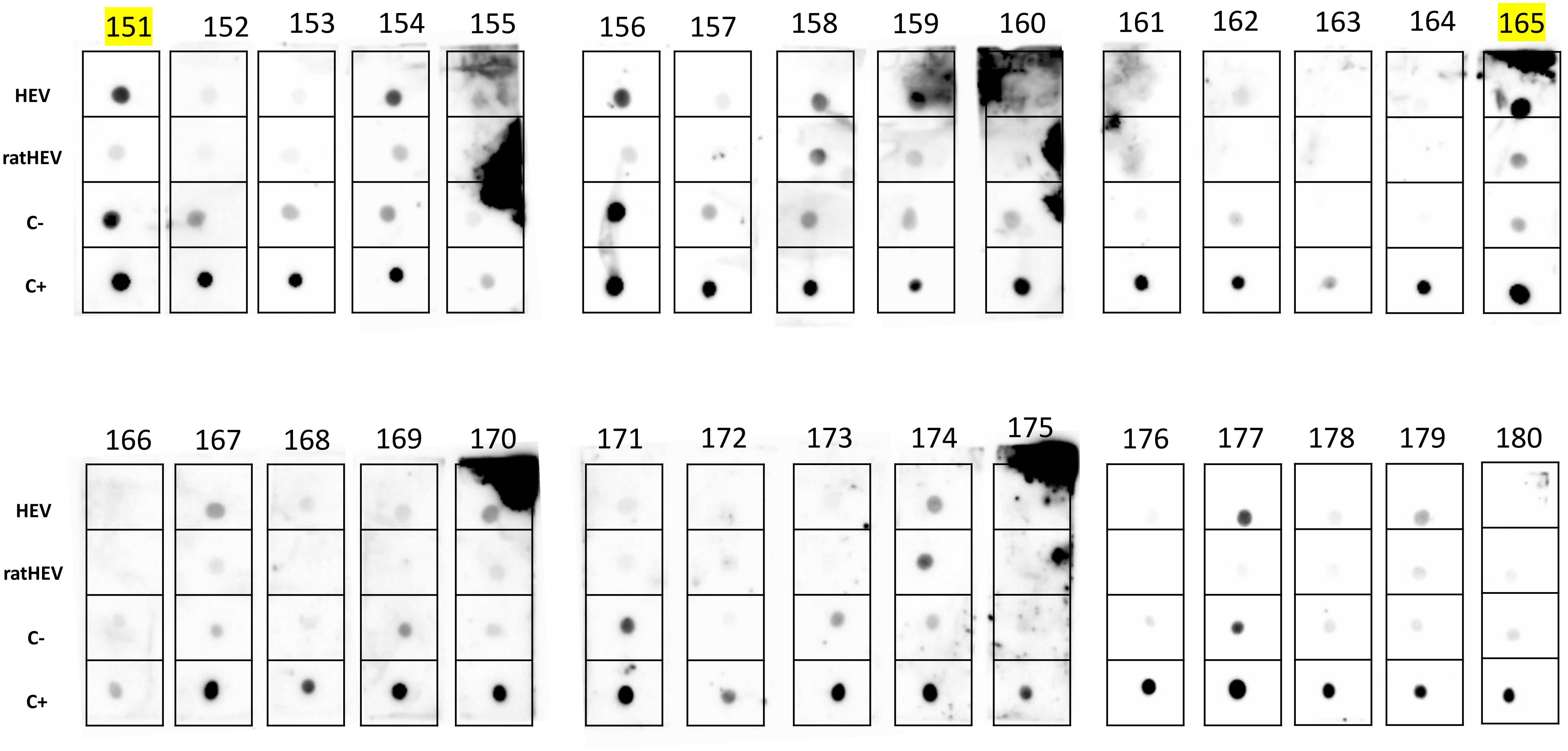
151
152
153
154
155
156
157
158
159
160
161
162
163
164
165
HEV
ratHEV
C-
C+
166
167
168
169
170
171
172
173
174
175
176
177
178
179
180
HEV
ratHEV
C-
C+

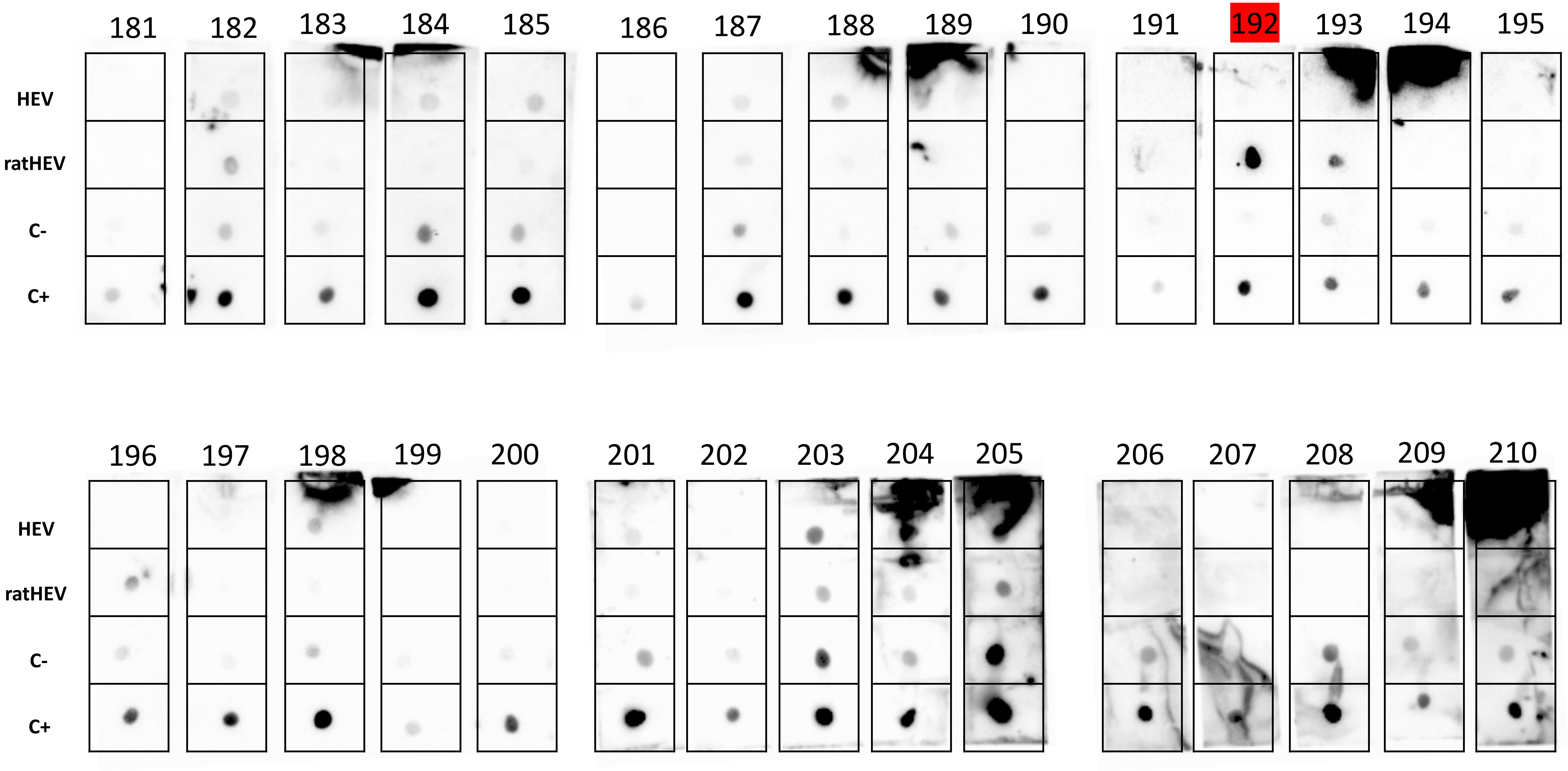
181
182
183
184
185
186
187
188
189
190
191
192
193
194
195
HEV
ratHEV
C-
C+
196
197
198
199
200
201
202
203
204
205
206
207
208
209
210
HEV
ratHEV
C-
C+

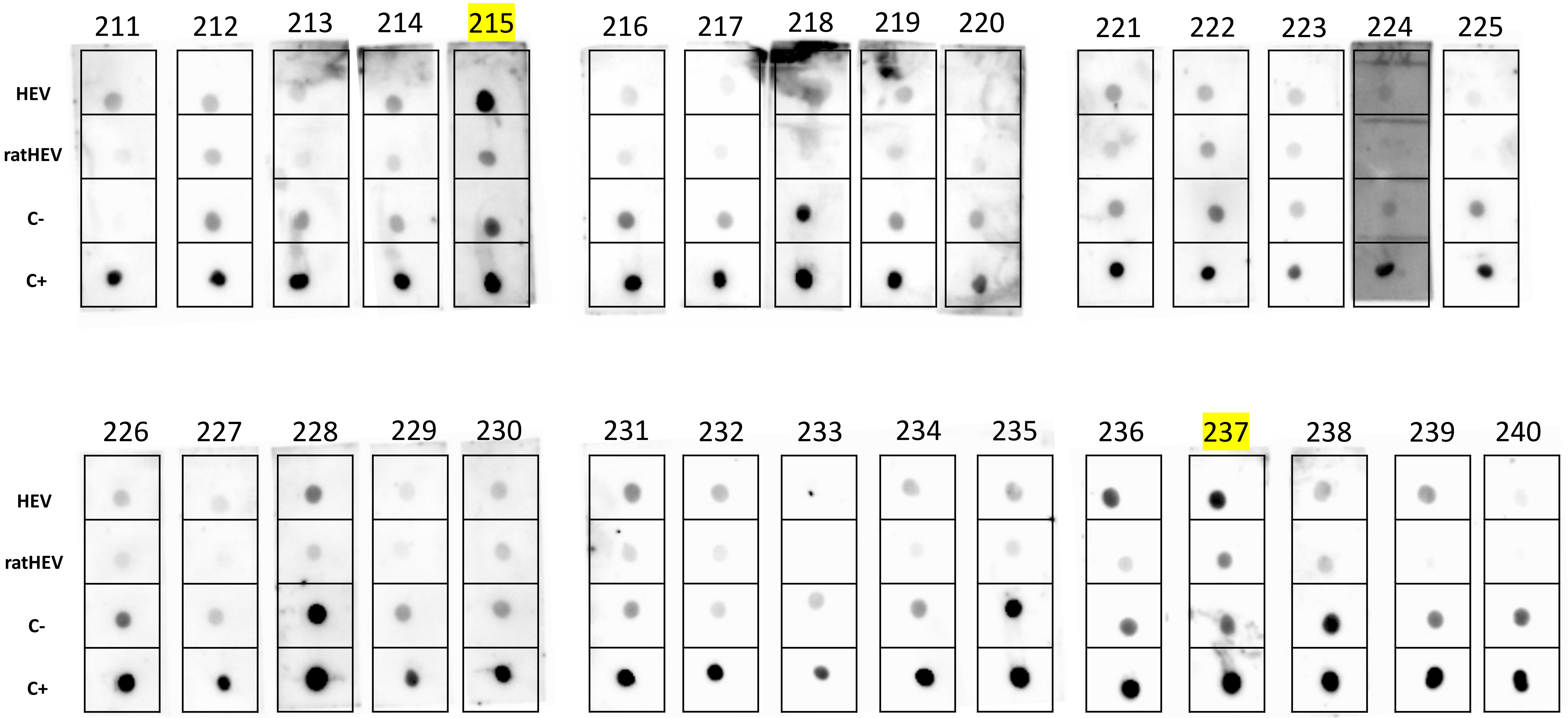
211
212
213
214
215
216
217
218
219
220
221
222
223
224
225
HEV
ratHEV
C-
C+
226
227
228
229
230
231
232
233
234
235
236
237
238
239
240
HEV
ratHEV
C-
C+

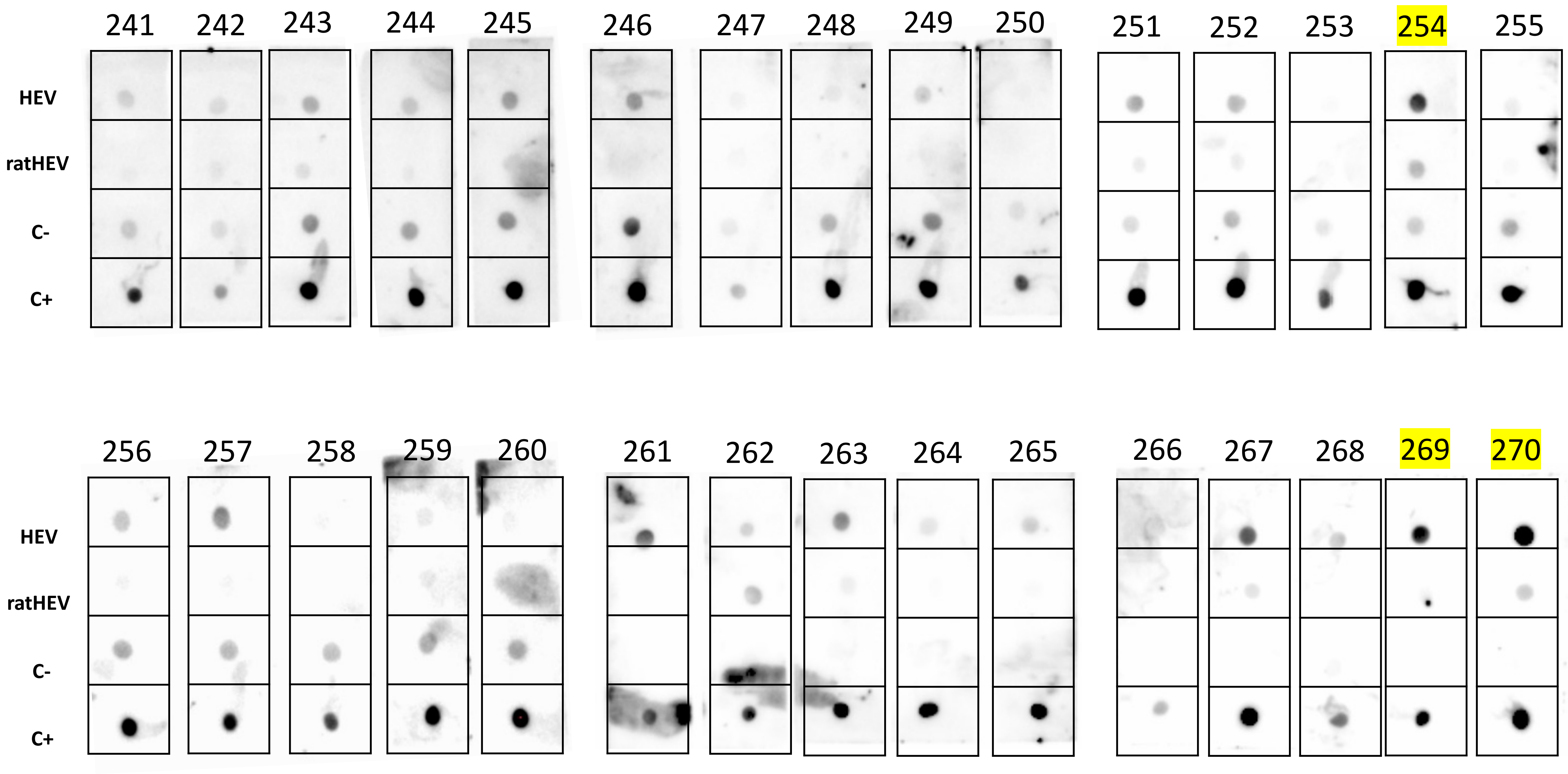
241
242
243
244
245
246
247
248
249
250
251
252
253
254
255
HEV
ratHEV
C-
C+
256
257
258
259
260
261
262
263
264
265
266
267
268
269
270
HEV
ratHEV
C-
C+

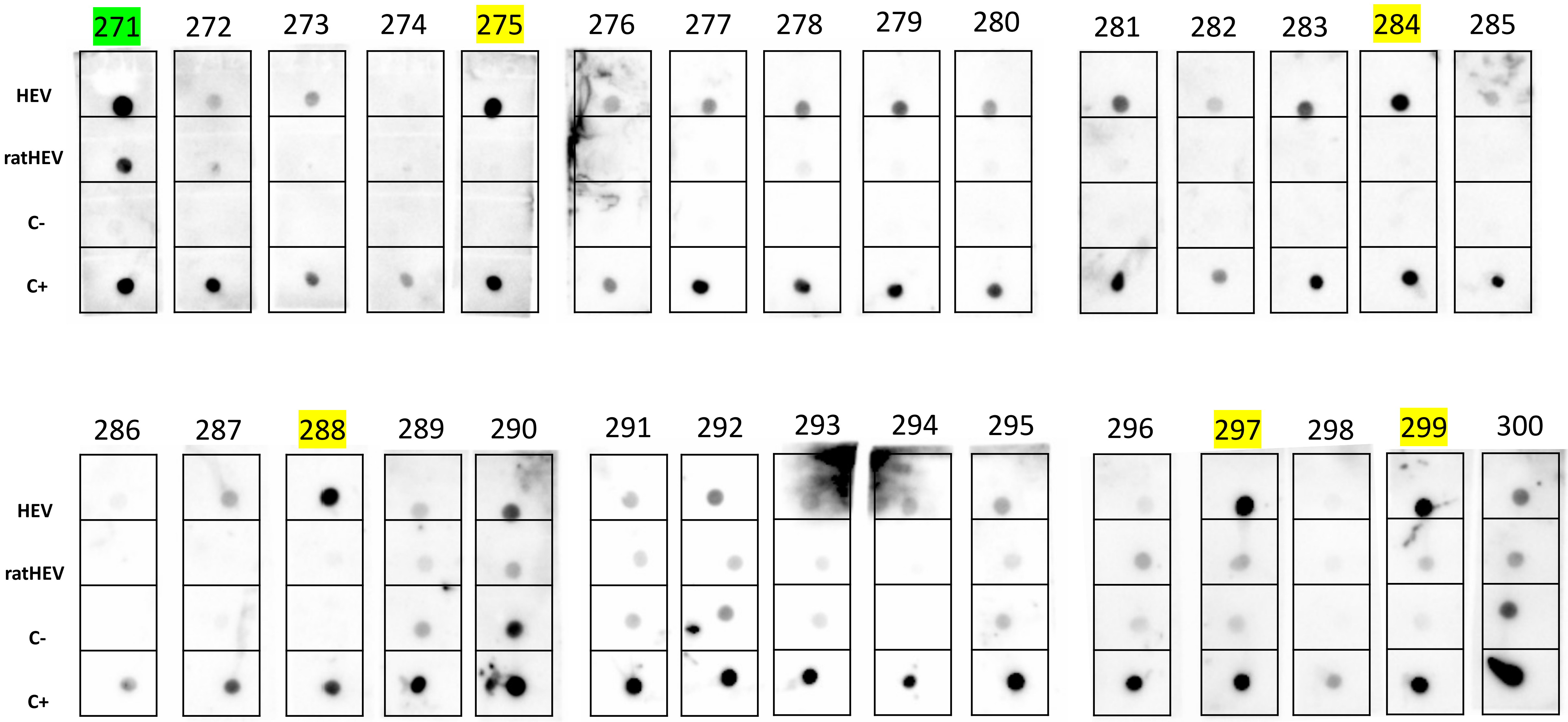

271
272
273
274
275
276
277
278
279
280
281
282
283
284
285
HEV
ratHEV
C-
C+
286
287
288
289
290
291
292
293
294
295
296
297
298
299
300
HEV
ratHEV
C-
C+

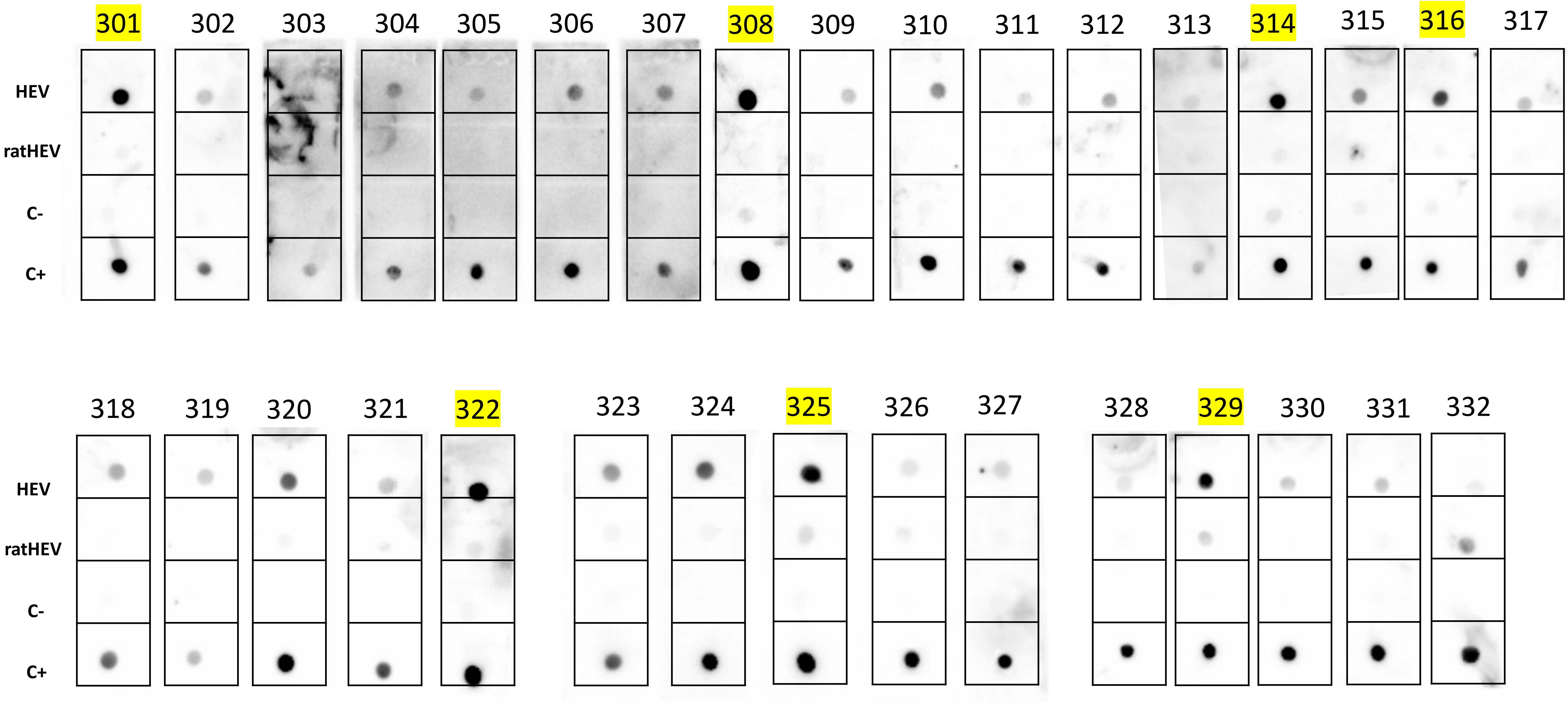

301
302
303
304
305
306
307
308
309
310
311
312
313
314
315
316
317
HEV
ratHEV
C-
C+
318
319
320
321
322
323
324
325
326
327
328
329
330
331
332
HEV
ratHEV
C-
C+

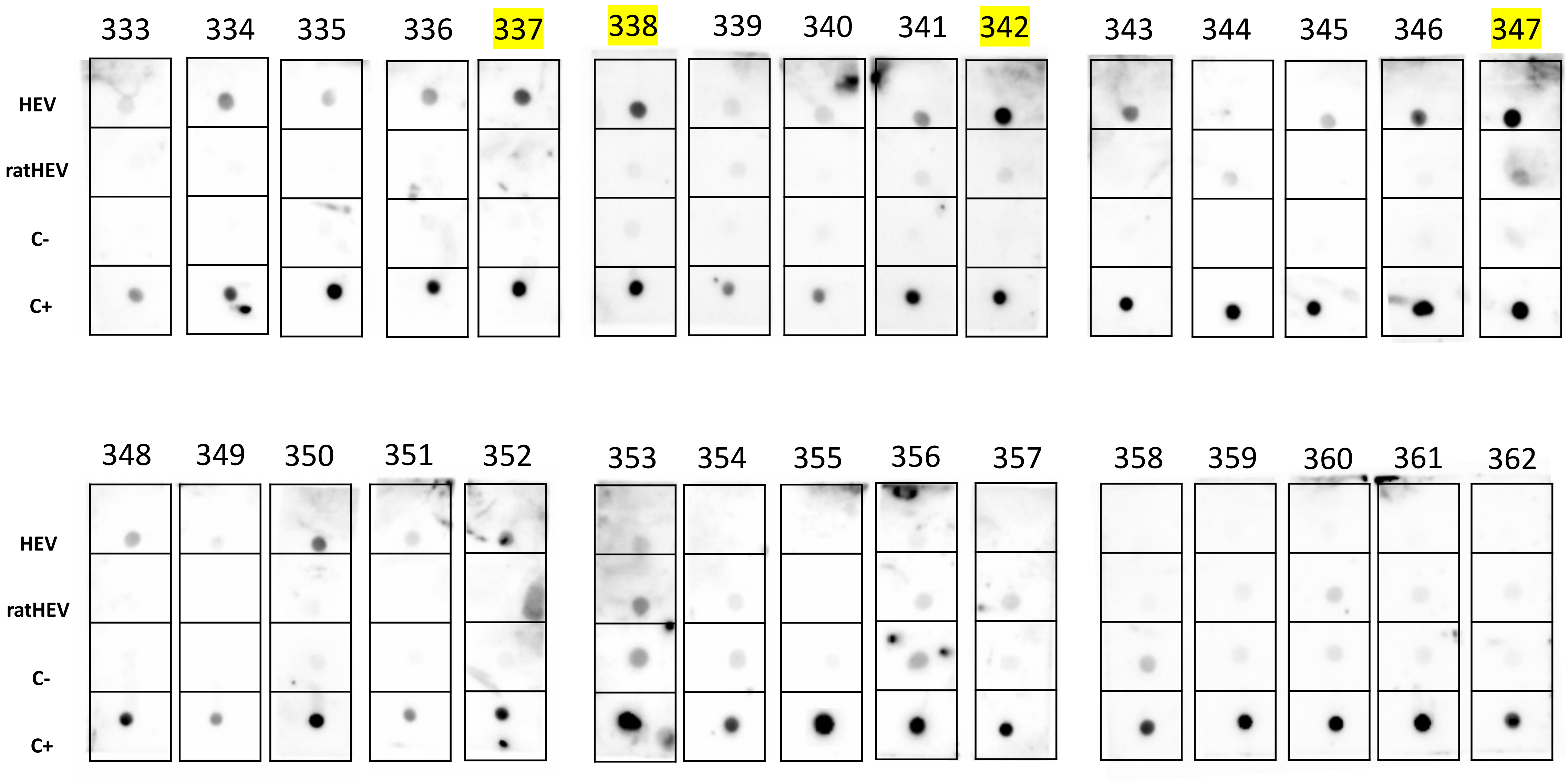
333
334
335
336
337
338
339
340
341
342
343
344
345
346
347
HEV
ratHEV
C-
C+
348
349
350
351
352
353
354
355
356
357
358
359
360
361
362
HEV
ratHEV
C-
C+

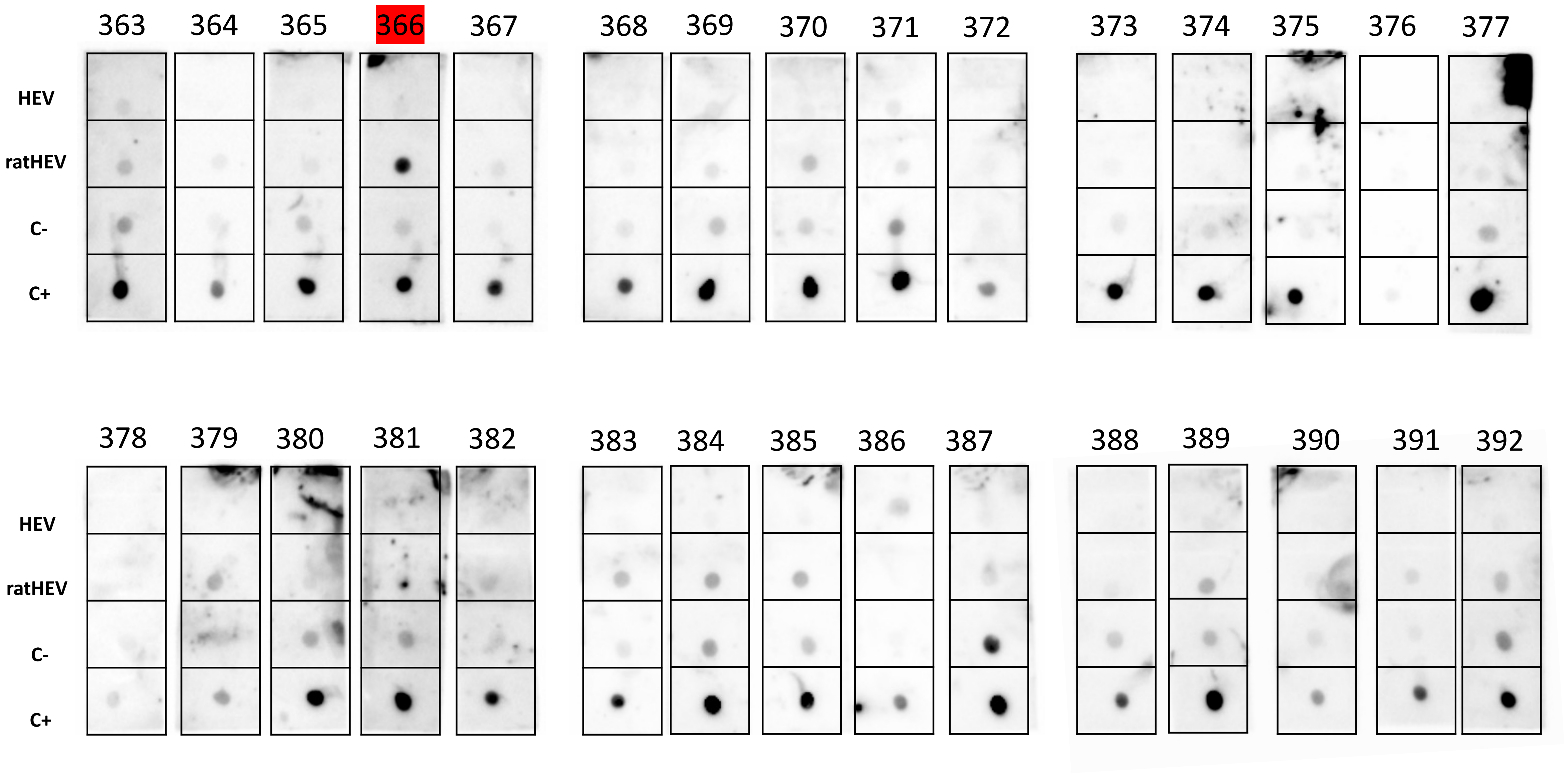
363
364
365
366
367
368
369
370
371
372
373
374
375
376
377
HEV
ratHEV
C-
C+
378
379
380
381
382
383
384
385
386
387
388
389
390
391
392
HEV
ratHEV
C-
C+

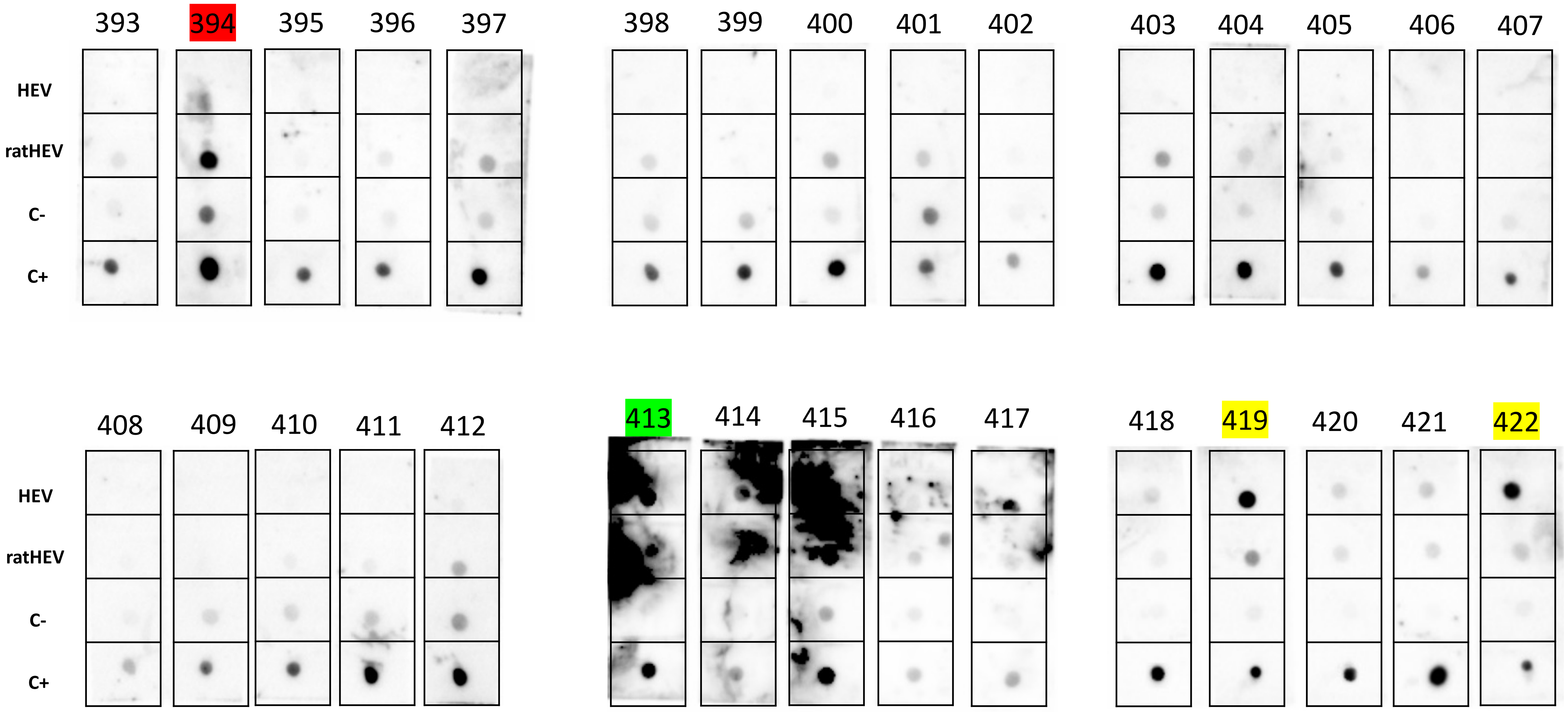
393
394
395
396
397
398
399
400
401
402
403
404
405
406
407
HEV
ratHEV
C-
C+
408
409
410
411
412
413
414
415
416
417
418
419
420
421
422

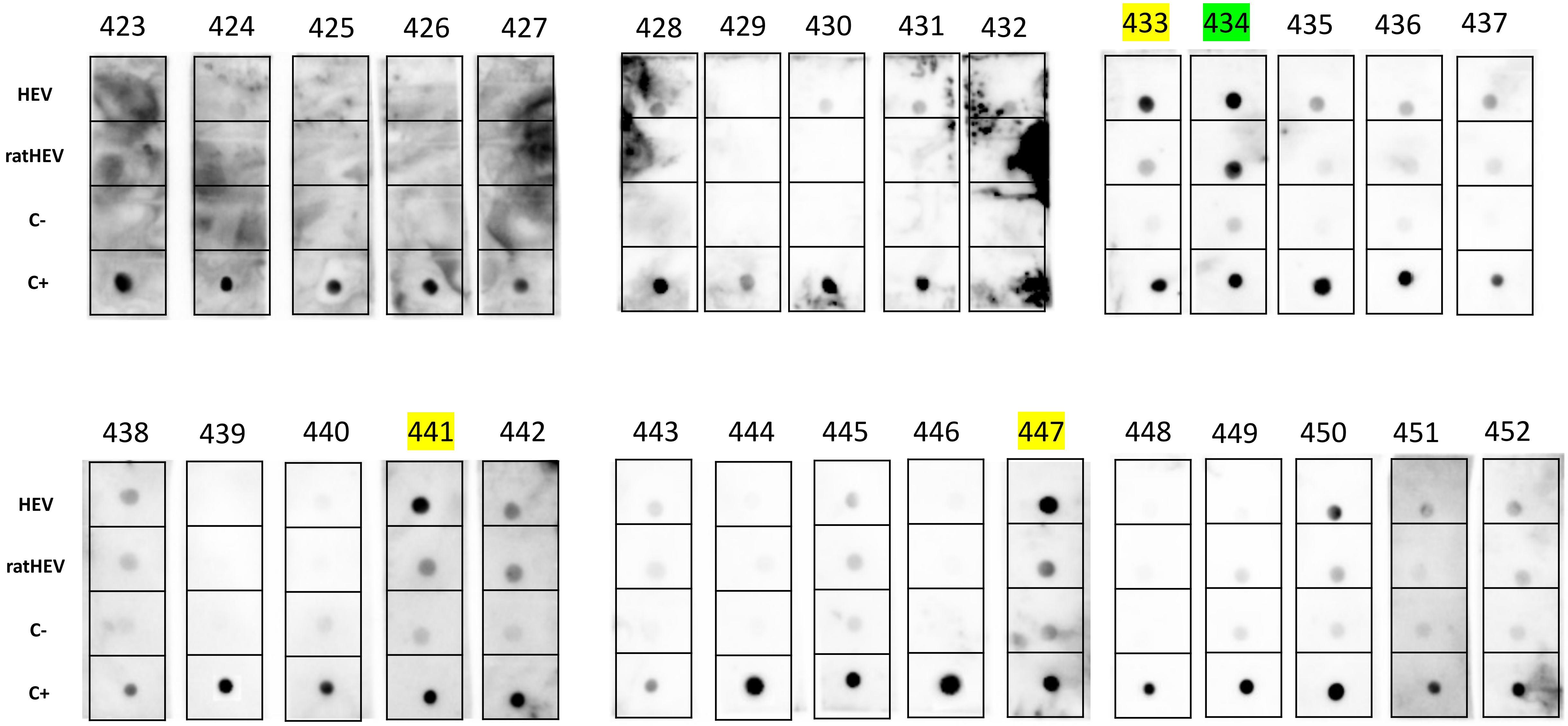

423
424
425
426
427
428
429
430
431
432
433
434
435
436
437
HEV
ratHEV
C-
C+
438
439
440
441
442
443
444
445
446
447
448
449
450
451
452
HEV
ratHEV
C-
C+

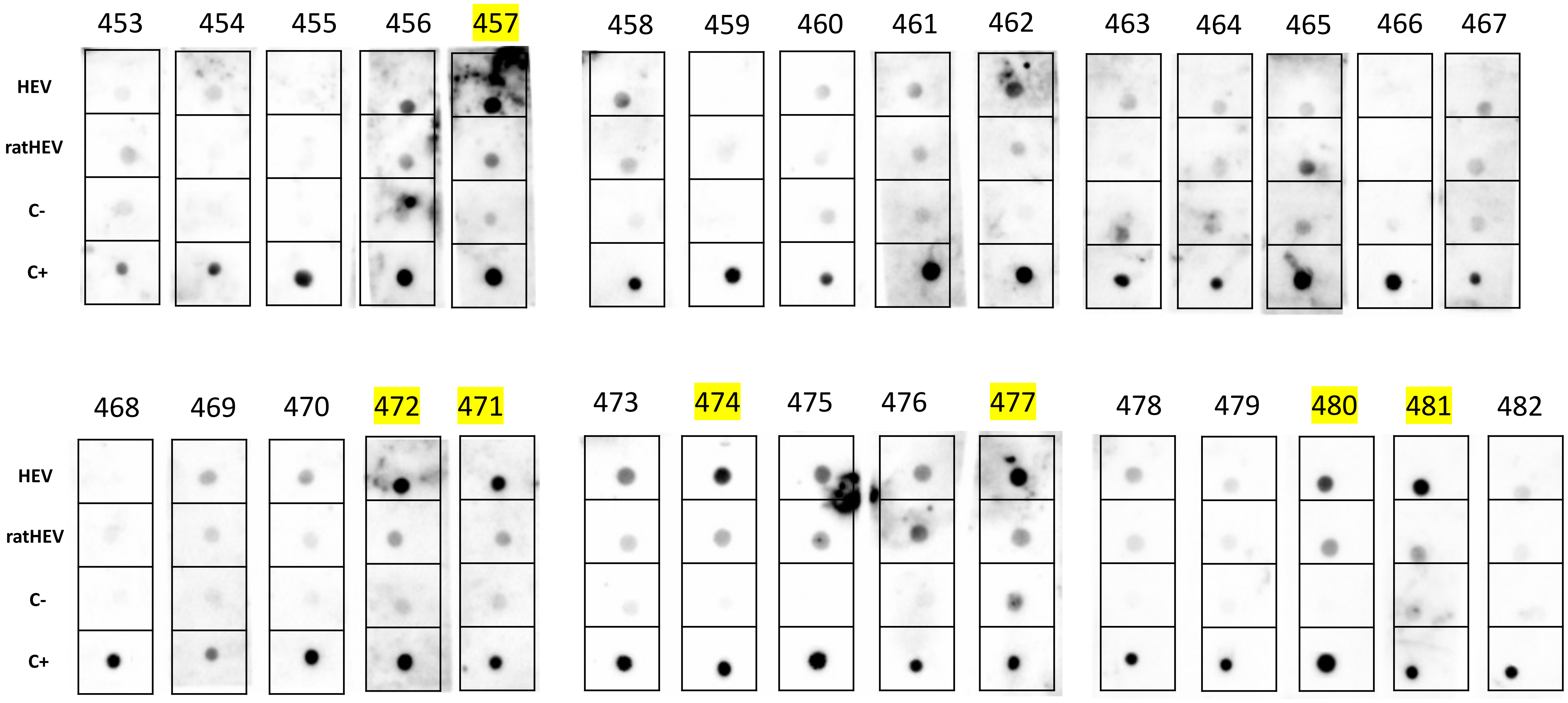
453
454
455
456
457
458
459
460
461
462
463
464
465
466
467
HEV
ratHEV
C-
C+
468
469
470
472
471
473
474
475
476
477
478
479
480
481
482
HEV
ratHEV
C-
C+

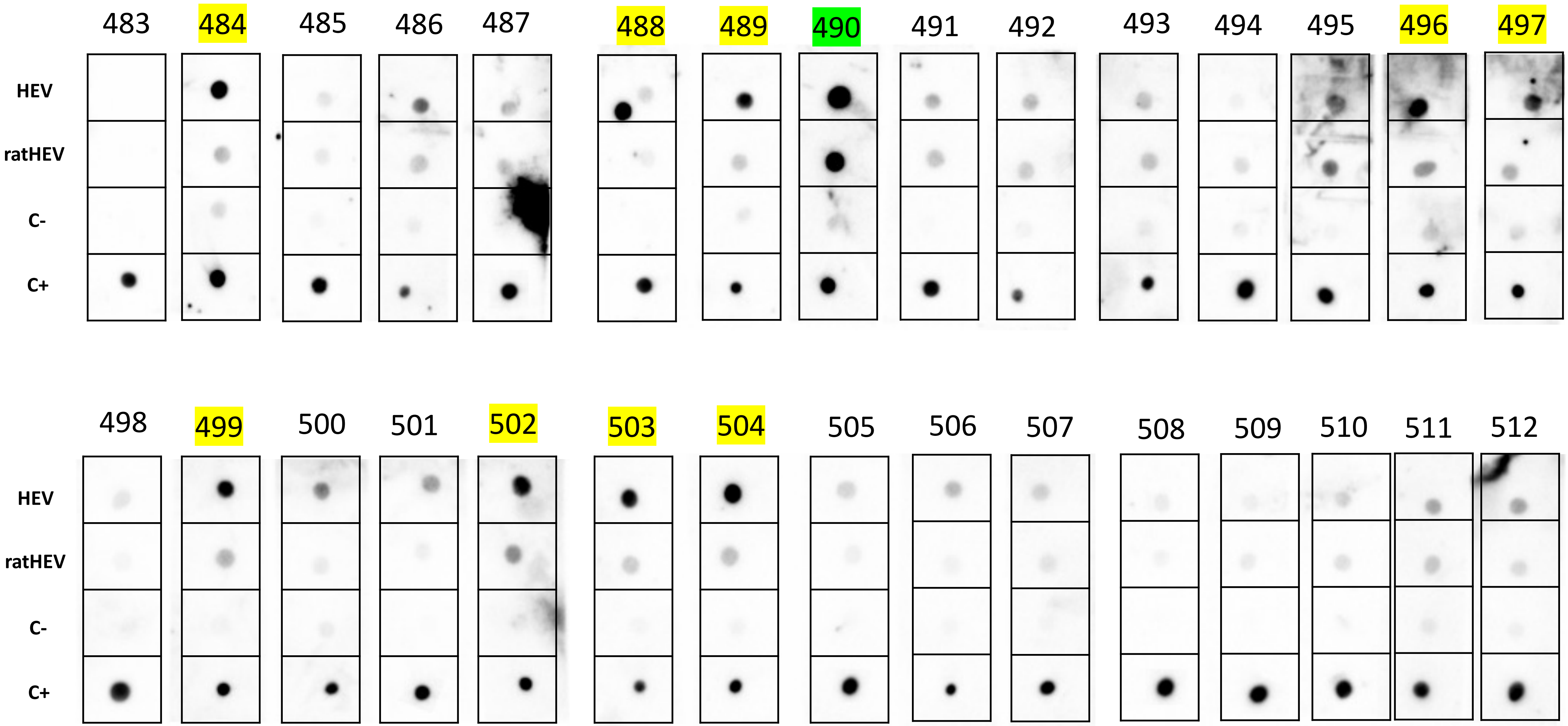
483
484
485
486
487
488
489
490
491
492
493
494
495
496
497
HEV
ratHEV
C-
C+
498
499
500
501
502
503
504
505
506
507
508
509
510
511
512
HEV
ratHEV
C-
C+

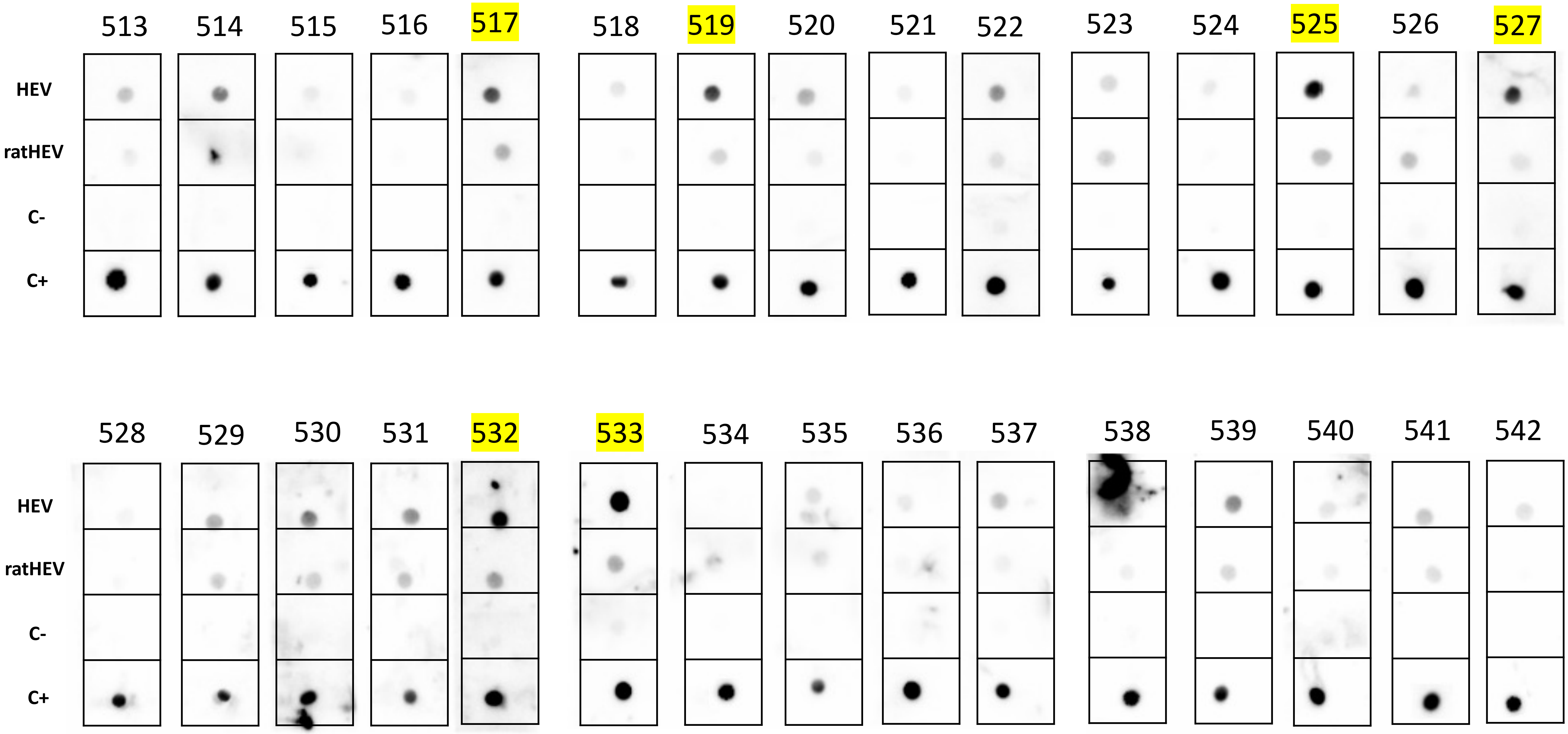
513
514
515
516
517
518
519
520
521
522
523
524
525
526
527
HEV
ratHEV
C-
C+
528
529
530
531
532
533
534
535
536
537
538
539
540
541
542
HEV
ratHEV
C-
C+

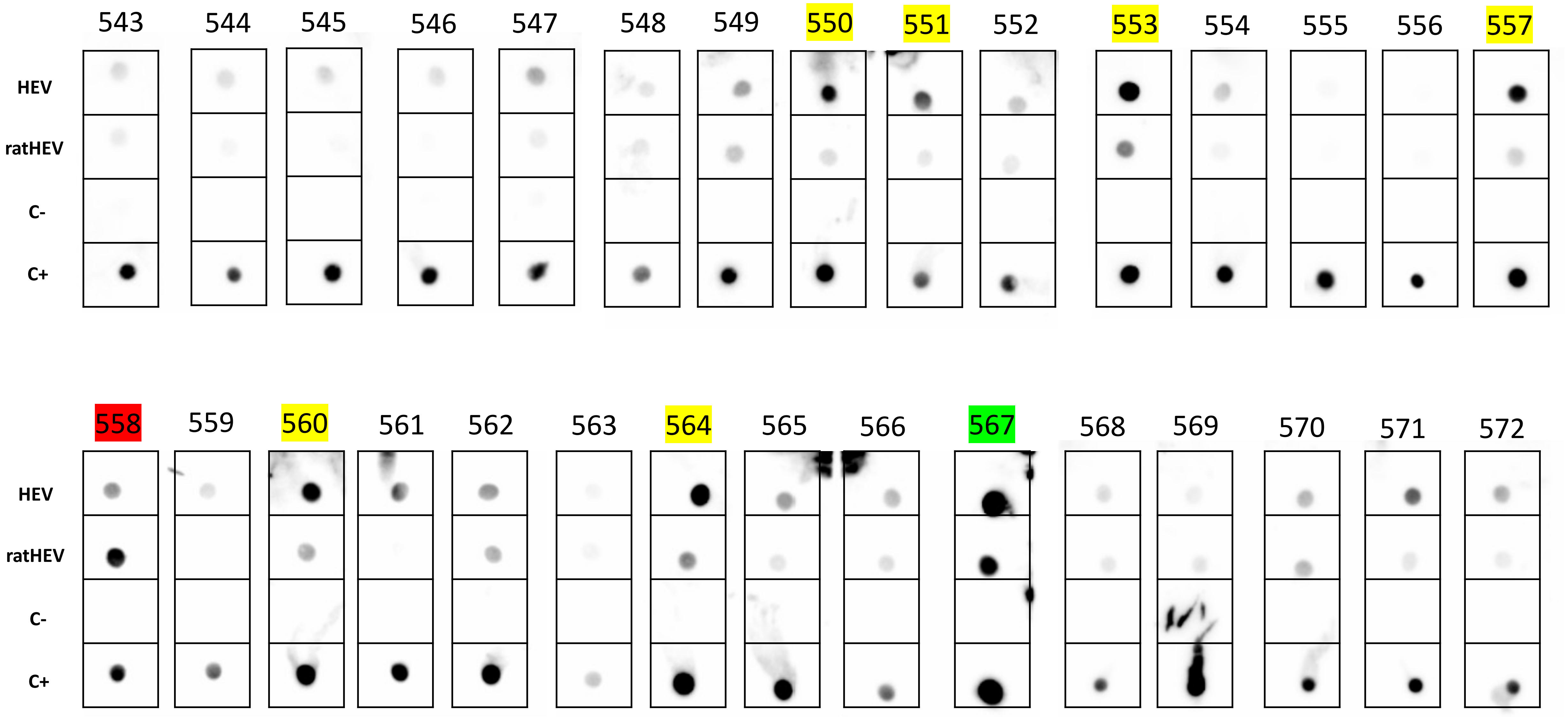
543
544
545
546
547
548
549
550
551
552
553
554
555
556
557
HEV
ratHEV
C-
C+
558
559
560
561
562
563
564
565
566
567
568
569
570
571
572
HEV
ratHEV
C-
C+

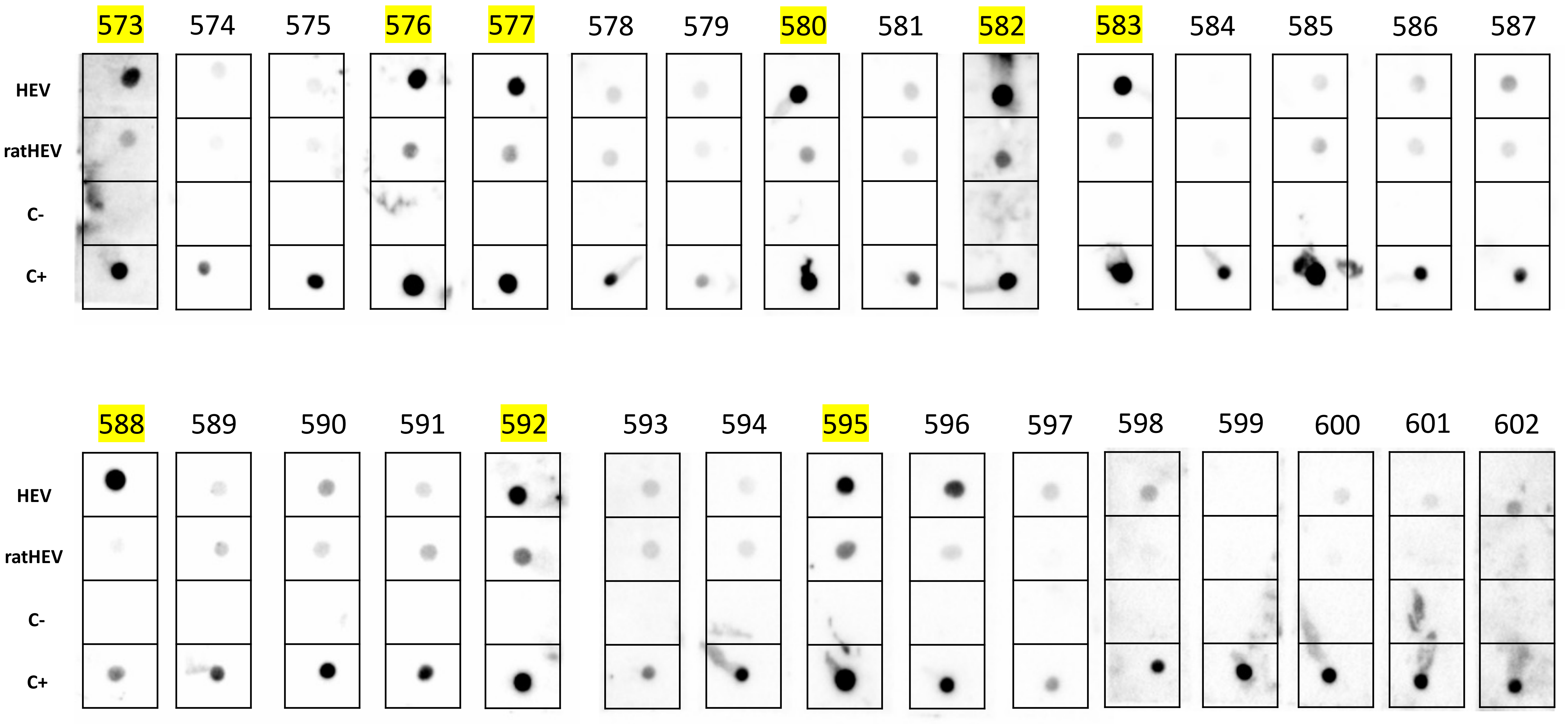
573
574
575
576
577
578
579
580
581
582
583
584
585
586
587
HEV
ratHEV
C-
C+
588
589
590
591
592
593
594
595
596
597
598
599
600
601
602
HEV
ratHEV
C-
C+

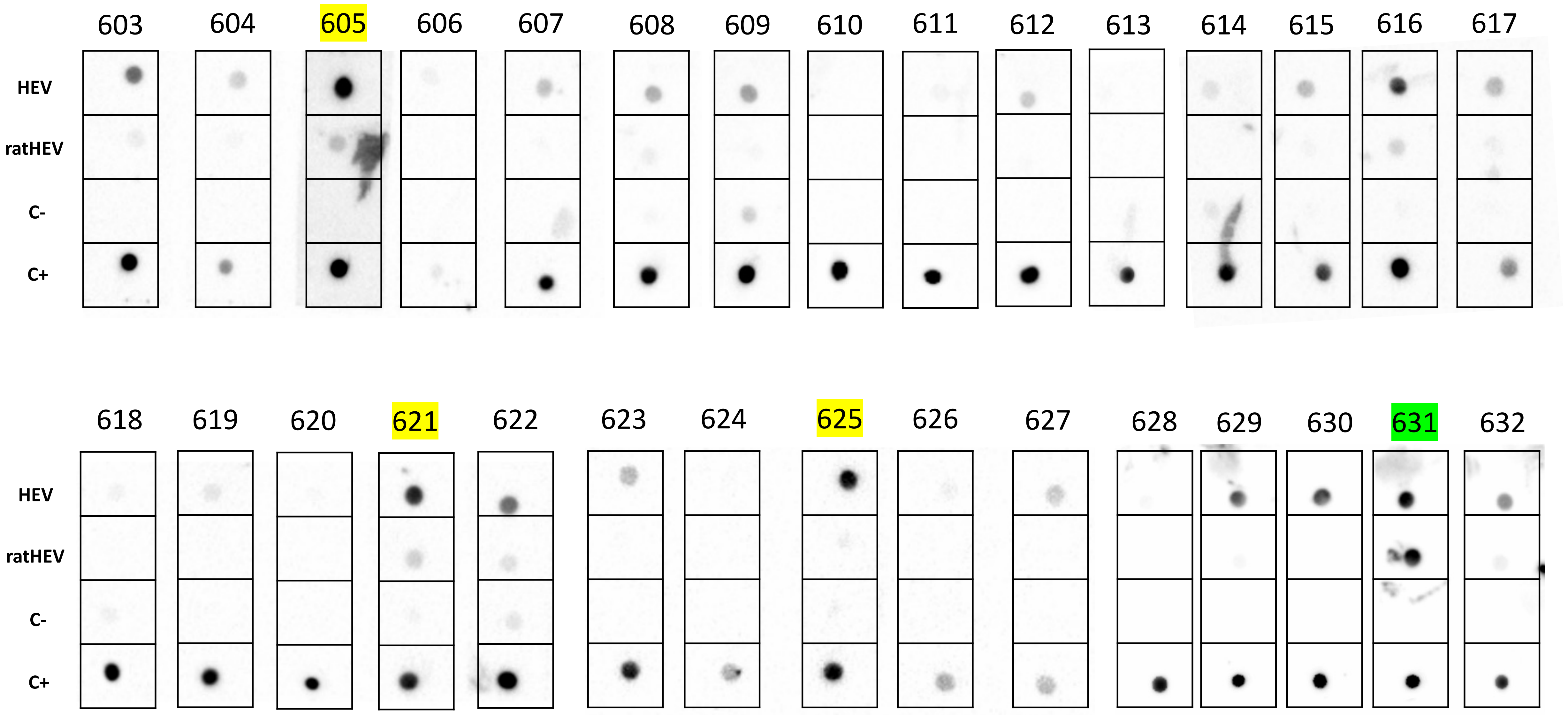
603
604
605
606
607
608
609
610
611
612
613
614
615
616
617
HEV
ratHEV
C-
C+
618
619
620
621
622
623
624
625
626
627
628
629
630
631
632
HEV
ratHEV
C-
C+

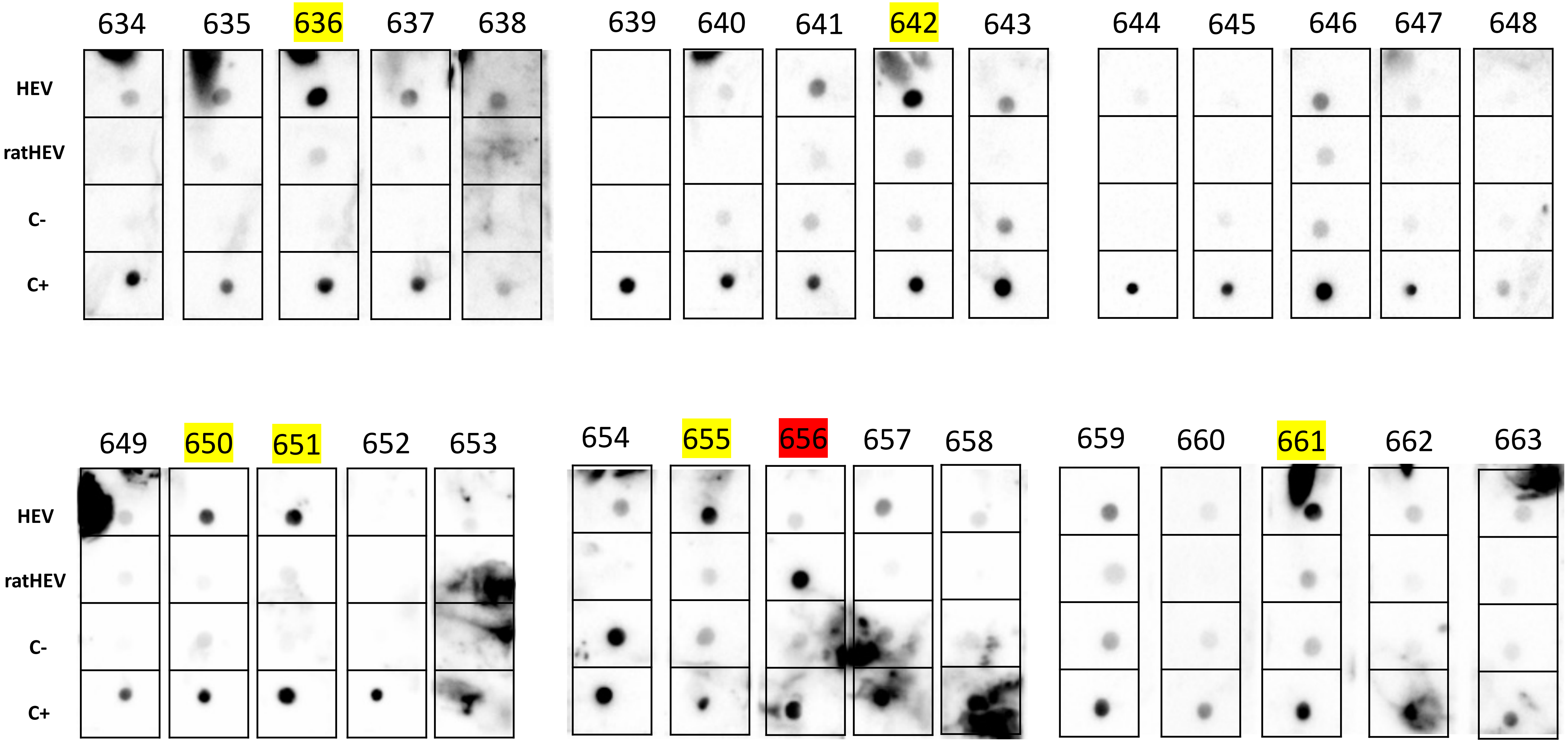
634
635
636
637
638
639
640
641
642
643
644
645
646
647
648
HEV
ratHEV
C-
C+
649
650
651
652
653
654
655
656
657
658
659
660
661
662
663
HEV
ratHEV
C-
C+

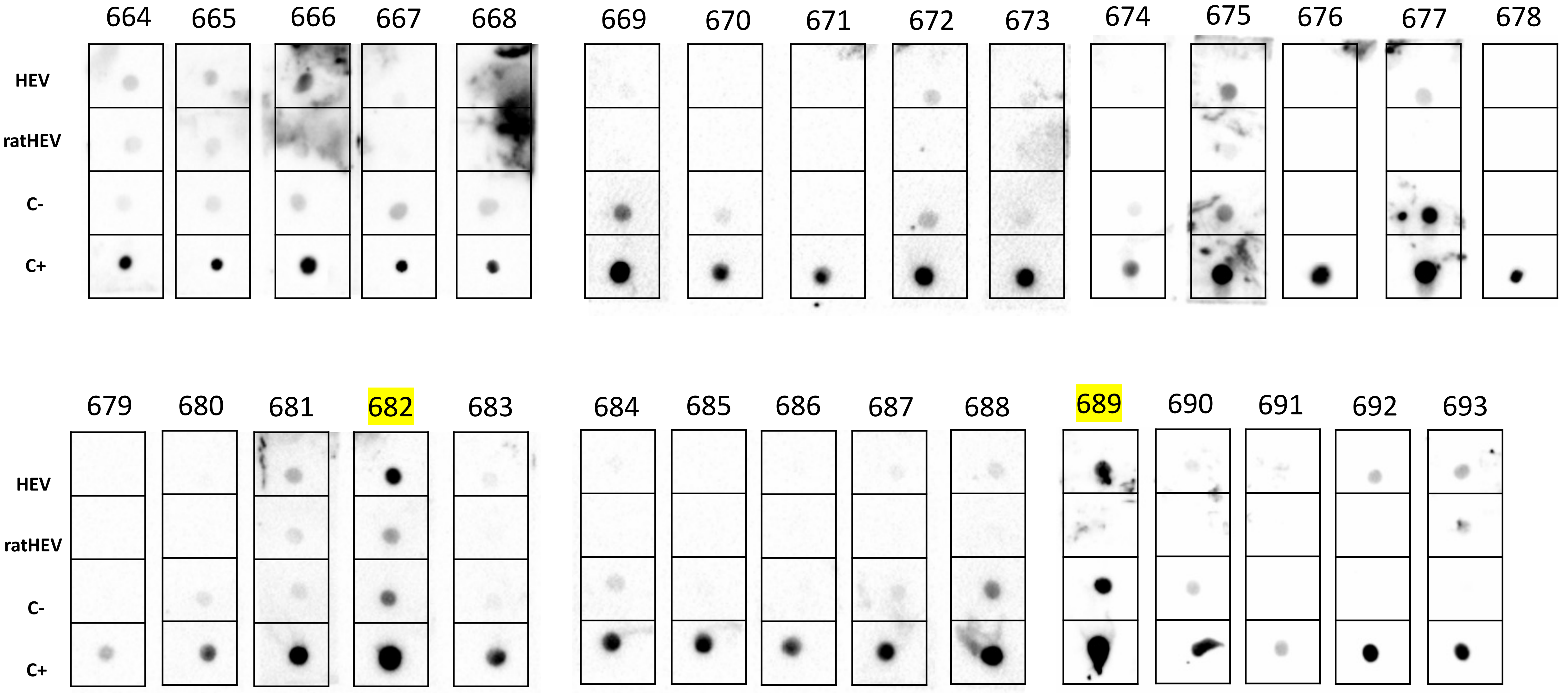

664
665
666
667
668
669
670
671
672
673
674
675
676
677
678
HEV
ratHEV
C-
C+
679
680
681
682
683
684
685
686
687
688
689
690
691
692
693
HEV
ratHEV
C-
C+

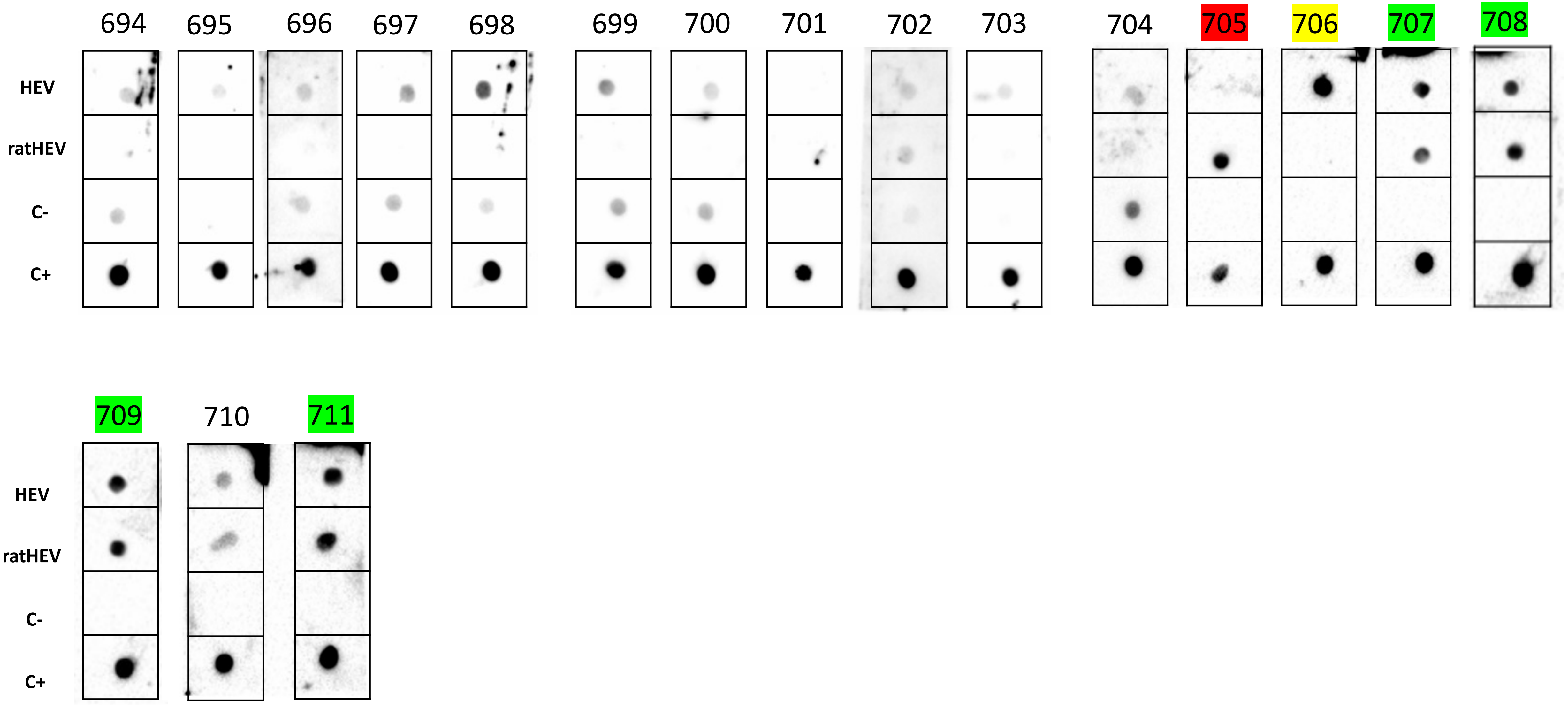

694
695
696
697
698
699
700
701
702
703
704
705
706
707
708
HEV
ratHEV
C-
C+
709
710
711
HEV
ratHEV
C-
C+

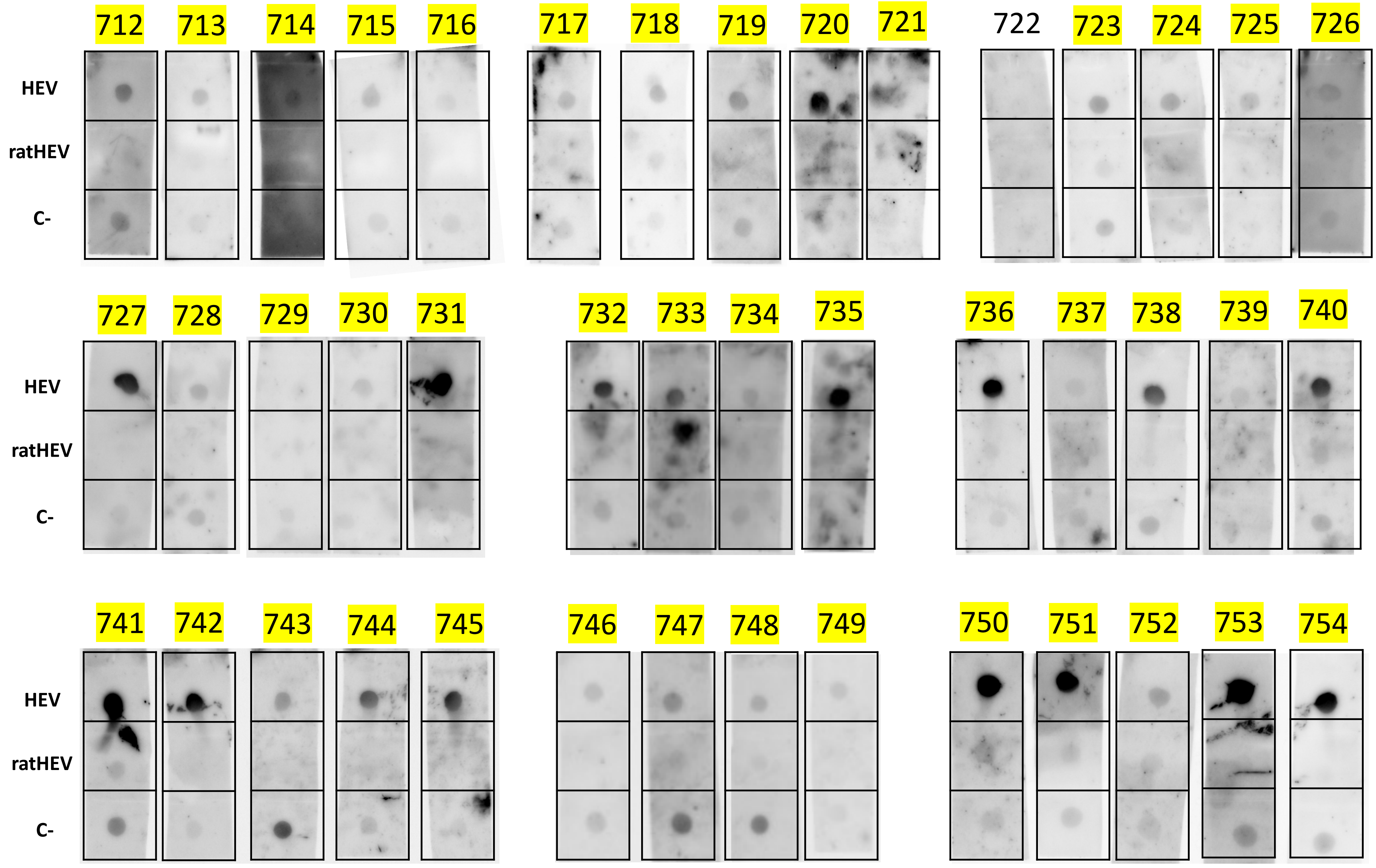
712
713
714
715
716
717
718
719
720
721
722
723
724
725
726
727
728
729
730
731
732
733
734
735
736
737
738
739
740
741
742
743
744
745
746
747
748
749
750
751
752
753
754
HEV
ratHEV
C-

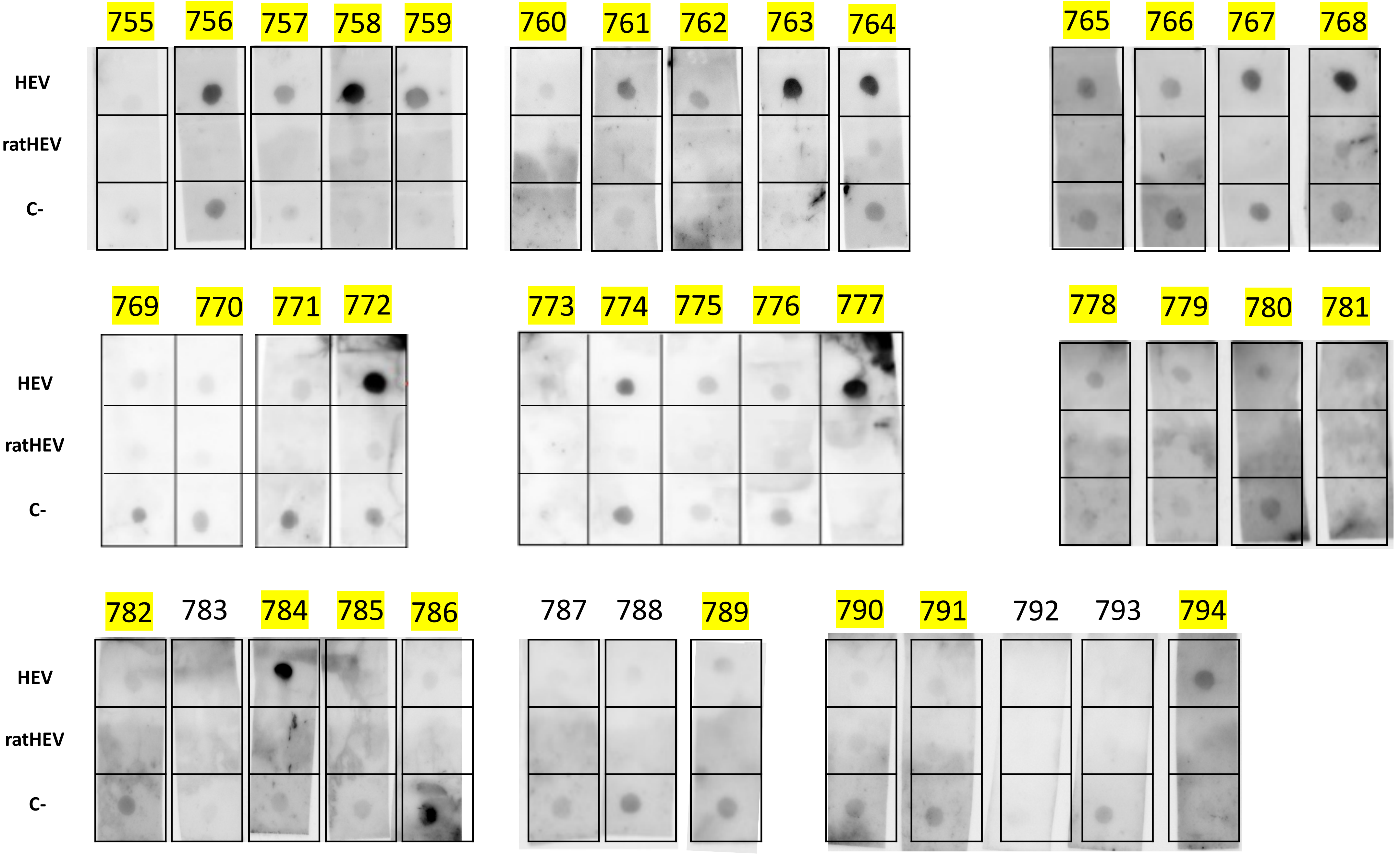
755
756
757
758
759
760
761
762
763
764
765
766
767
768
HEV
ratHEV
C-
769
770
771
772
773
774
775
776
777
778
779
780
781
HEV
ratHEV
C-
782
783
784
785
786
787
788
789
790
791
792
793
794
HEV
ratHEV
C-

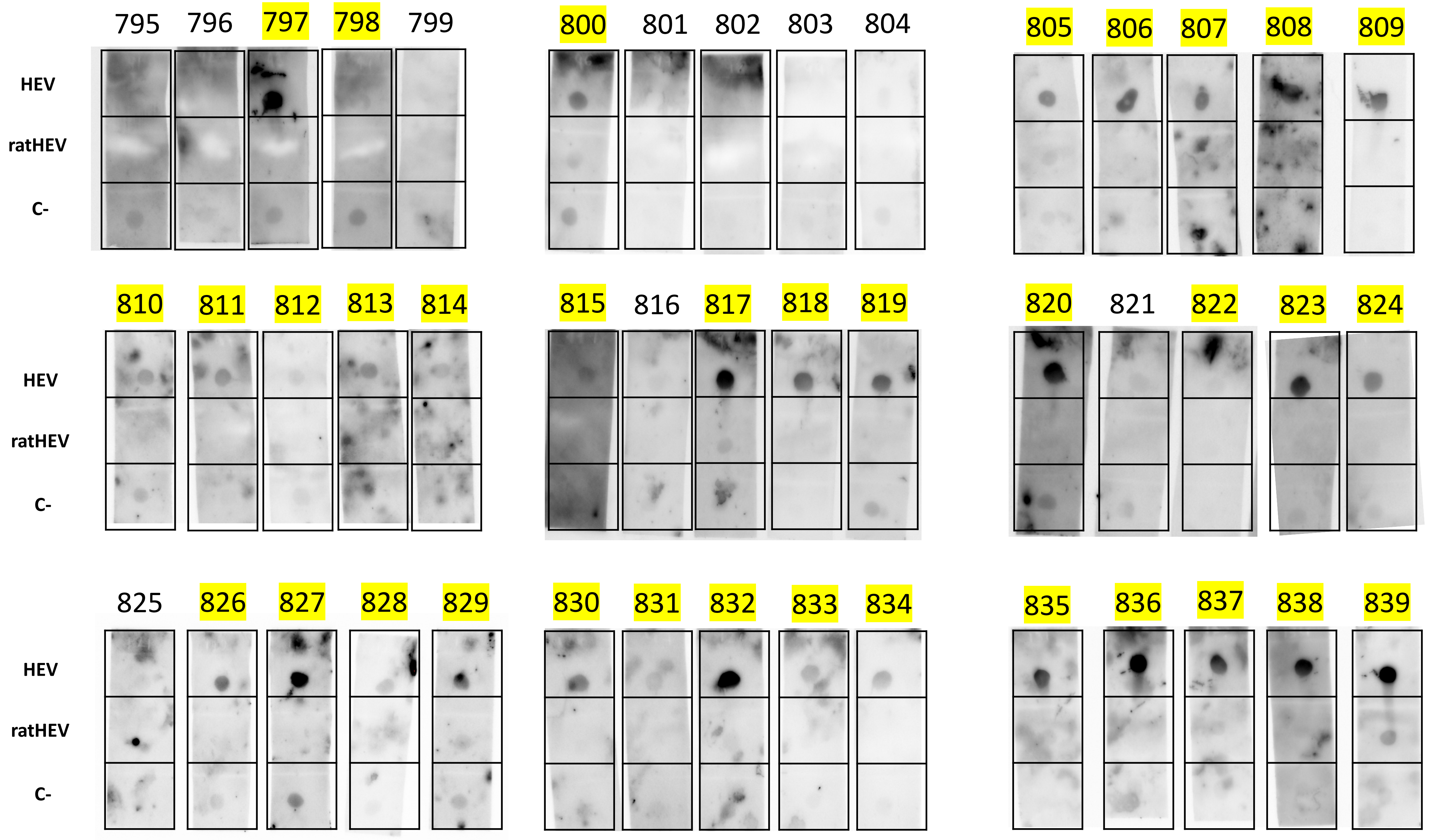
795 796 797 798 799
800 801 802 803 804
805 806 807 808 809
810 811 812 813 814
815 816 817 818 819
820 821 822 823 824
825 826 827 828 829
830 831 832 833 834
835 836 837 838 839
HEV
ratHEV
C-

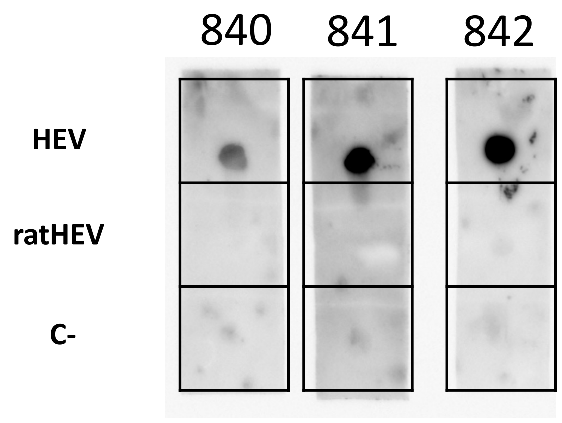

840
841
842
HEV
ratHEV
C-
